# Hepatitis C Virus-Induced Cytoplasmic Organelles Use the Nuclear Transport Machinery to Establish an Environment Conducive to Virus Replication

**DOI:** 10.1371/journal.ppat.1003744

**Published:** 2013-10-31

**Authors:** Christopher J. Neufeldt, Michael A. Joyce, Aviad Levin, Rineke H. Steenbergen, Daniel Pang, Justin Shields, D. Lorne J. Tyrrell, Richard W. Wozniak

**Affiliations:** 1 Department of Cell Biology, University of Alberta, Edmonton, Alberta, Canada; 2 Department of Medical Microbiology and Immunology, University of Alberta, Edmonton, Alberta, Canada; University of California, San Diego, United States of America

## Abstract

Hepatitis C virus (HCV) infection induces formation of a membranous web structure in the host cell cytoplasm where the viral genome replicates and virions assemble. The membranous web is thought to concentrate viral components and hide viral RNA from pattern recognition receptors. We have uncovered a role for nuclear pore complex proteins (Nups) and nuclear transport factors (NTFs) in the membranous web. We show that HCV infection leads to increased levels of cytoplasmic Nups that accumulate at sites enriched for HCV proteins. Moreover, we detected interactions between specific HCV proteins and both Nups and NTFs. We hypothesize that cytoplasmically positioned Nups facilitate formation of the membranous web and contribute to the compartmentalization of viral replication. Accordingly, we show that transport cargo proteins normally targeted to the nucleus are capable of entering regions of the membranous web, and that depletion of specific Nups or Kaps inhibits HCV replication and assembly.

## Author Summary

Hepatitis C virus (HCV) is a positive strand RNA virus and is a major cause of liver disease worldwide, affecting more than 170 million individuals. Infection of cells with HCV leads to rearrangement of cytoplasmic host cell membranes into viral replication and assembly complexes collectively known as the membranous web. This membranous web is thought to be involved in concentrating viral components and immune evasion, though the mechanisms by which these functions are achieved remains an important question in the field. Here, we report that nuclear envelope structures that transport macromolecules into and out of the nucleus, termed nuclear pore complexes (NPCs), are also present in the membranous web of cells infected with HCV and other positive strand RNA viruses. Our results suggest that these NPCs function to regulate access of proteins into the interior of the membranous web, thus contributing to the establishment of an environment conducive to viral replication and viral immune evasion. Consistent with this idea, we show that NPC proteins are required for HCV assembly. Our discovery that nuclear transport proteins play a role in HCV replication, and potentially other viral infections, may lead to the discovery of new targets for antiviral therapies.

## Introduction

Hepatitis C virus (HCV) is a positive strand RNA virus of the *Flaviviradae* family, a blood borne pathogen and a major cause of liver disease worldwide, with an estimated 170 million people infected [1]. Approximately 30% of chronically infected patients develop progressive liver disease, including cirrhosis and end stage liver disease [2]. HCV is now the leading indication for liver transplantation in North America [3].

Recruitment and rearrangement of host cell membranes has been observed during the life cycle of numerous viruses including hepatitis B virus, cytomegalovirus, and all positive strand RNA viruses [4], [5], [6], [7]. In HCV-infected cells, these membrane structures, termed the membranous web, consist of numerous double membrane vesicles, as well as multivesicular units and lipid droplets surrounded by membranes [8], [9], [10], which arise primarily from the endoplasmic reticulum (ER) as well as from other membranes derived from the secretory pathway. The membranous web constitutes a virally-induced organelle that has been shown to be a separate compartment from the cytoplasm [11], [12]. Though the precise structure and function of the membranous web remains unclear, it is proposed to have a variety of functions including viral packaging and egress, and concentration and synchronization of viral replication and assembly. In addition, it has been proposed to facilitate avoidance of host cell cytoplasmic pattern recognition receptors (PRRs) [5], [13].

Owing to their small genome size, some viruses hijack host proteins for their own purposes. However, in the case of HCV, which does not have an obvious nuclear component to its life cycle, it is difficult to reconcile the number of viral interactions with components of the nuclear transport machinery [14], [15], [16], [17], [18], [19], [20]. These nuclear transport components include soluble nuclear transport factors (NTFs), many of which are members of a family of proteins termed karyopherins (Kaps). Kaps bind nuclear localization/import signal (NLS) or nuclear export signal (NES) containing molecules in the cytoplasm or nucleus and escort these cargos across the nuclear envelope (NE) through passageways formed by large macromolecular structures termed nuclear pore complexes (NPCs) (reviewed in [21]). Each NPC is comprised of ∼30 distinct proteins, called nucleoporins (Nups), that form a cylindrical channel lined by Nups that facilitate movement of the NTF across the nuclear envelope. Studies examining a number of viruses have reported interactions between viral proteins and NTFs and/or Nups. In some cases, these interactions support nuclear functions of viral proteins or act to alter host cell nuclear transport [22], [23], [24]. However, in situations where the virus life cycle has no clear nuclear intermediate, such as with HCV, the function of viral protein interactions with NTFs or Nups is unclear [18], [19], [25]. For example, four of the ten HCV proteins have been shown to contain putative NLS sequences, and can enter the nucleus when mutated or produced outside of the context of viral infection, but only the core protein has been suggested to enter the nucleus of HCV-infected hepatocytes ([15], [17], [25], [26]; unpublished data).

We have investigated the potential functions of Nups and NTFs in supporting HCV infection. Initially, we monitored the consequences of HCV infection on Nups. Following HCV infection, we observed an increase in cytoplasmic levels of various Nups and their recruitment to regions of cytoplasm containing HCV replication or assembly complexes. Consistent with these observations, we show an association between various HCV proteins and specific Nups, as well as the NTFs Kap β3/IPO5 and Kap α. These interactions appear to play a key role in the viral life cycle, as depletion of specific Nups or Kap β3/IPO5 inhibits HCV replication. Furthermore, we present data that support a model in which the critical function of the nuclear transport components in HCV replication and assembly occurs in the cytoplasm where they contribute to the structure and function of the membranous web.

## Results

### HCV recruits Nups to sites of viral assembly

Observations that several HCV proteins, such as core and NS5A, interact with nuclear transport factors are perplexing, given that these proteins are membrane associated and detected in the cytoplasm, where they participate in HCV replication or assembly. Core, for example, is detected in distinct regions of the cytoplasm and associated with membranes surrounding lipid droplets (Figure S1A) [10]. These regions of core concentration lie primarily within areas of the cytoplasm that contain the membranous web [10]. Consistent with this concept, regions of the cytoplasm containing the bulk of the core protein appear largely devoid of microtubules, presumably being excluded by the membranous web (Figure S1B). NS5A and NS3 are also detected in the membranous web [10] and in these regions of microtubule exclusion (Figure S1C, and data not shown). However, like several nonstructural proteins, NS5A and NS3 exhibit a broader cytoplasmic distribution outside the membranous web owning to their presence within the ER [10], [27].

**Figure 1:**
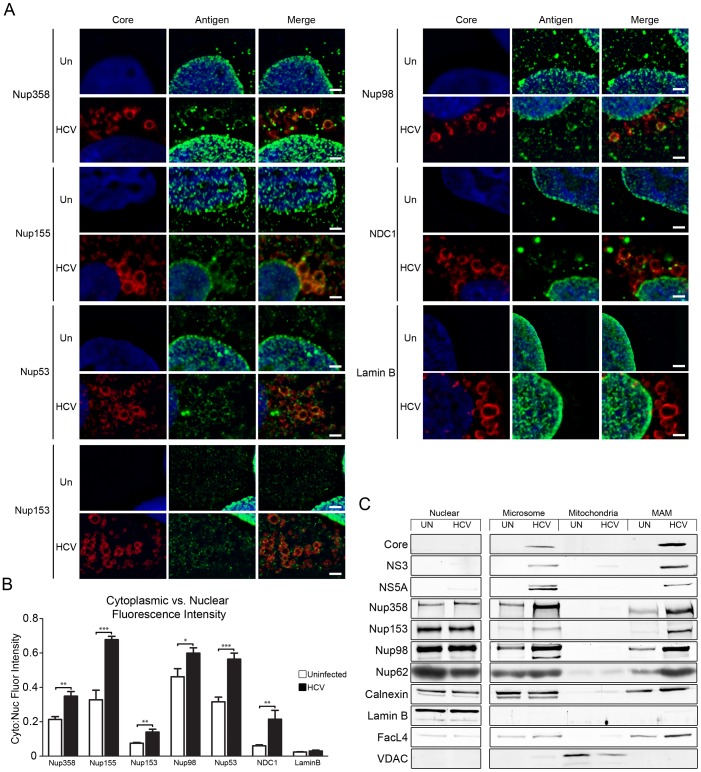
Cytoplasmic localization of Nups in HCV-infected tissue culture cells. Huh7.5 cells were uninfected or infected with HCV for four days. A) Localization of Nups in cells either uninfected (Un) or HCV-infected (HCV) was evaluated by indirect immunofluorescence microscopy using antibodies specific for the indicated Nups or lamin B (green) and HCV core (red). DNA was stained with DAPI (blue). Scale bar, 2 µm (A). B) Changes in the cytoplasmic levels of Nups induced by HCV infection were quantified. The ratio of cytoplasmic to nuclear fluorescence levels were calculated for the indicated Nups and lamin B in uninfected and HCV-infected cells (n≥10). The statistical significance of differences between uninfected and infected ratios was determined (p-values less than 0.05 (*), 0.01 (**) and 0.001 (***) are indicated). C) Total cell lysates were isolated from uninfected (UN) or infected (HCV) Huh7.5 cells and subjected to subcellular fractionation. Western blotting using antibodies specific for the indicated proteins was used to evaluate the protein levels in each of the indicated subcellular fractions. In addition to the Nups and HCV proteins indicated, markers for residue ER (calnexin), NE (lamin B), mitochondria (VDAC), and MAM (FacL4) proteins are shown. Equal amounts of total protein were loaded into each lane. All samples were run on the same gel and images shown are derived from scans of the same membrane.

We postulated that interactions between HCV components and the nuclear transport machinery could contribute to cytoplasmic processes. Cytoplasmic functions for Kaps have been documented [28], and in many cell types a population of NPCs (termed annulate lamellae) are present in the endoplasmic reticulum (ER) where they form transcisternal pores across parallel ER membranes similar to those in the NE. These cytoplasmic NPCs are transport competent, however, what roles they play are unknown [29], [30]. We hypothesized that, during HCV infection, cytoplasmic NPCs might function in the membranous web. To investigate this idea, we first examined subcellular localization of various Nups in Huh7.5 cells infected HCV genotype 2a strain JFH-1. In uninfected cells, immunofluorescence microscopy analysis using antibodies directed against various Nups, including Nup358, Nup155, Nup53, Nup153, Nup98, and NDC1, an integral membrane component of NPCs, revealed a punctate NE pattern representative of NPCs and cytoplasmic foci characteristic of annulate lamellae (Figure 1A). In HCV-infected cells, a similar NE signal was observed, however cytoplasmic levels of each Nup were increased (Figure 1A, 1B, and S2A). Strikingly, the cytoplasmic Nup signals often colocalized with core protein, notably around lipid droplets, and in regions of the cytoplasm with reduced microtubules and containing NS5A-positive membranes (Figure 1A, S2B, S4, and S6). A spatial relationship between core and the Nups was further demonstrated by line graphs of fluorescence intensity through regions containing lipid droplets (Figure S3). Interestingly, this redistribution of Nups to cytoplasmic compartments was also observed in cells infected other positive-strand RNA viruses, including Hepatitis A virus and Dengue virus, suggesting that there may be a conserved role for cytoplasmic Nups in positive-strand RNA virus infection (Figure S5).

We further evaluated the consequences of HCV infection on Nup localization and the physical proximity of Nups and HCV proteins using subcellular fractionation. Subcellular fractionation procedures have previously detected HCV proteins in membranes fractions with sedimentation characteristics similar to microsomes and mitochondrial-associated membranes (MAM) [31], [32], [33]. We also detected an enrichment of HCV proteins in similar membrane fractions but not in more rapidly sedimenting nuclei and mitochondria containing fractions (Figure 1C). In uninfected cells, Nups are primarily detected in nuclear fractions, with the lower levels of these proteins detected in microsomes and the more rapidly sedimenting MAM fraction likely arising from annulate lamellae. Consistent with our immunofluorescence microscopy analysis showing a close association of Nups with core and the membranous web, we observed that HCV infected cells contained increased amounts of various Nups in the microsomal and MAM fractions together with core and the non-structural proteins NS3 and NS5A (Figure 1C). By contrast, Nup amounts in nuclear fractions were unchanged in the HCV infected cells as compared to their uninfected counterparts. Barely detectable amounts of lamin B are seen in the MAM fractions of infected cells, perhaps reflecting a minor nuclear contamination of these fractions or the binding of lamin B to the Nups in these fractions. Nuclear contamination does not explain the increased levels of Nups in the MAM fraction as the Nup∶lamin B signal ratio is strikingly higher in this fraction as compared to the nuclear fraction.

**Figure 2:**
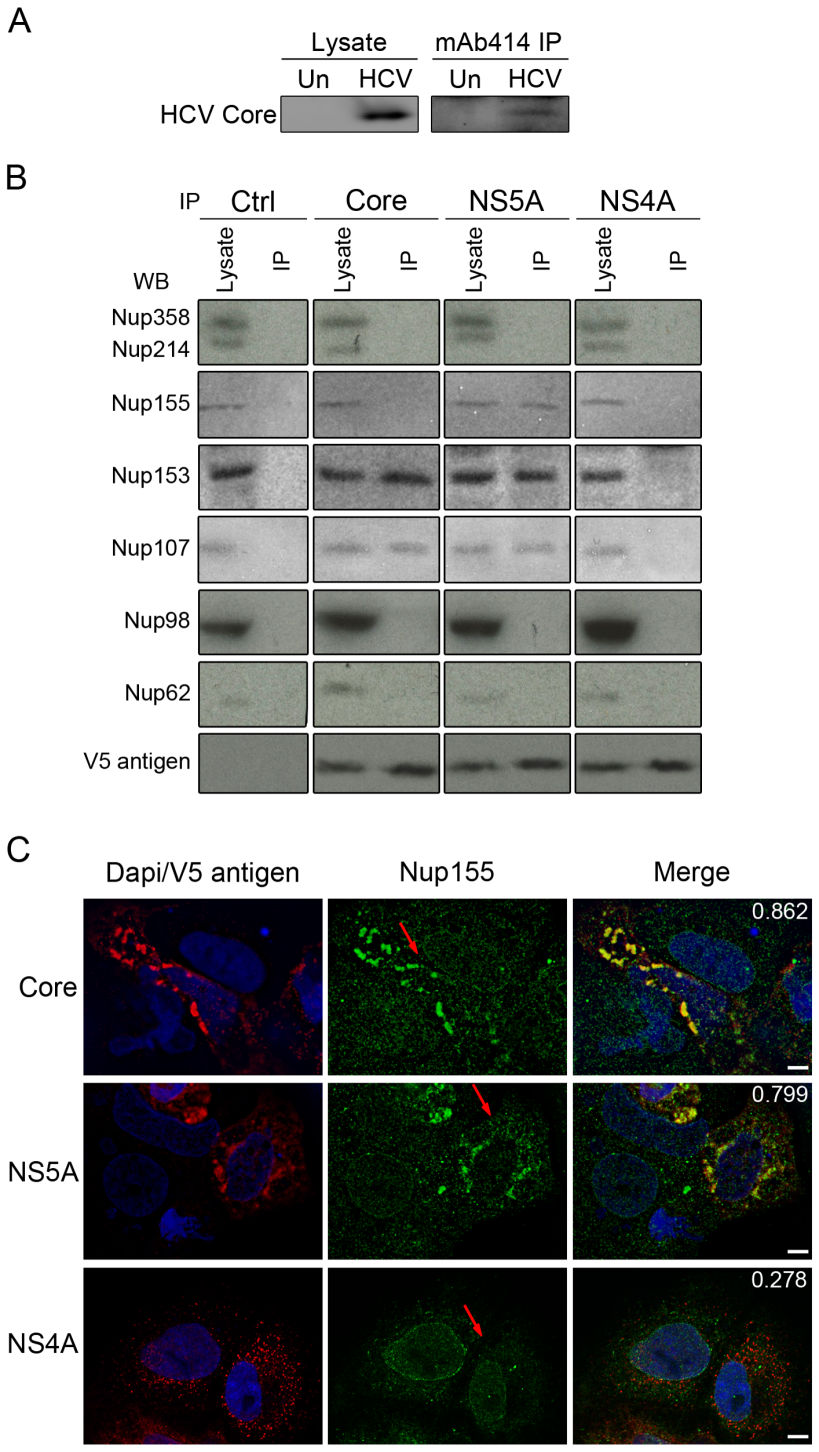
Identification of Nups that physically interact with HCV proteins. A) Cell lysates were isolated from uninfected (Un) and HCV-infected (HCV) Huh7.5 cells four days after infection. The nuclear fraction was removed from the lysates by sedimentation and the remaining cytoplasmic fraction was incubated with the monoclonal antibody mAb414 specific for a subset of Nups. Proteins present in cell lysates and mAb414 immunoprecipitates (mAb414 IP) were analyzed by western blotting using antibodies specific for HCV. B) Constructs encoding for the indicated V5-tagged HCV proteins or an empty V5 vector (ctrl) were transfected into HEK293T cells and expressed for 48 hrs. V5-tagged proteins were immunoprecipitated with anti-V5 antibodies and associated proteins were evaluated by western blotting using antibodies against the indicated Nups and the V5 epitope. C) Subcellular localization of Nup155 in Huh7.5 cells expressing the indicated V5-tagged HCV proteins was examined by indirect immunofluorescence microscopy using antibodies directed against Nup155 (green) and the V5 epitope (red). DNA is visualized with DAPI (blue) and arrows point to transfected cells. Pearson's colocalization coefficients were calculated to assess colocalization of Nup155 and the indicated V5 tagged protein and are shown at the top of the merged panel. Values >0.5 are considered significant. Scale bar, 10 µm.

To investigate the molecular basis for the interactions of HCV core and other viral proteins with NPCs, we performed immunoprecipitation experiments. Two approaches were used to assess HCV protein-Nup interactions. To examine the interactions of core with NPCs, Nups present in a post-nuclear supernatant derived from HCV infected cell lysates were immunoprecipitated using a monoclonal antibody (mAb414) that binds a shared epitope present in several Nups, including Nup358, Nup214, Nup153, and Nup62. Consistent with our immunofluorescence results, western analysis of the immunopurified Nups detected associated core protein (Figure 2A). Similarly, we examined whether immune-purified HCV proteins were bound to Nups. As the purification of HCV proteins from infected cells was unsuccessful, we chose to introduce genes encoding individual HCV proteins tagged with a V5 epitope into HEK293T cells and immunoprecipitate the tagged proteins using anti-V5 antibodies. Western analysis of immunopurified core, NS5A, and NS4A detected Nups associated with a subset of these proteins (Figure 2B). Nup107 and Nup153, components of the NPC scaffold and attached filaments, were detected in association with HCV core and NS5A, while Nup358, Nup214, Nup98, and Nup62 were not detected in these immunoprecipitates. Another component of the NPC scaffold, Nup155, was detected in association with NS5A but not with the core protein. By contrast, we failed to detect any Nups bound to immunoprecipitated NS4A.

The interactions of core and NS5A with Nups were further validated by immunofluorescence analysis of cells expressing genes encoding these tagged HCV proteins or the JFH-1 subgenomic replicon. In cells producing core or NS5A, regions of the cytoplasm containing these proteins also showed colocalizing Nups, including Nup155 and Nup98 (Figure 2C and S6A); moreover, these Nups appeared reduced at the NE. A similar phenotype was not observed in cells expressing NS4A, leading us to conclude that core and NS5A are among those HCV proteins that interact with Nups. We also examined the localization of several Nups in cell expressing the JFH-1 subgenomic replicon, which lacks the coding region for core through the NS2 protein of the HCV polyprotein. Cells containing the replicon develop membrane alterations similar to the HCV-induced membranous-web [34]. In these cells we detected increased cytoplasmic levels of Nup358 and extensive colocalization of Nup155 with membrane-associated NS5A (Figure S7). Consistent with these observations, in HCV infected cells, cytoplasmic Nup155 exhibited an ∼55% overlap with NS5A (Figure S6B and S6C), and, more generally, the increased cytoplasmic NPC foci seen during infection occupy similar regions of the cytoplasm as membranous web-associated NS5A (as revealed using anti-Nup98 antibodies; Figure S5B). Interestingly, some Nups that were recruited to the cytoplasmic membranes in the HCV infected cells were not altered in their distribution in the replicon containing cells, including Nup98 and NDC1 (Figure S7). These results imply that those HCV proteins missing from the replicon containing cells, such as core, are required for the recruitment of additional Nups to cytoplasmic membranes.

### HCV infection alters Nup mRNA and protein levels

**Figure 3:**
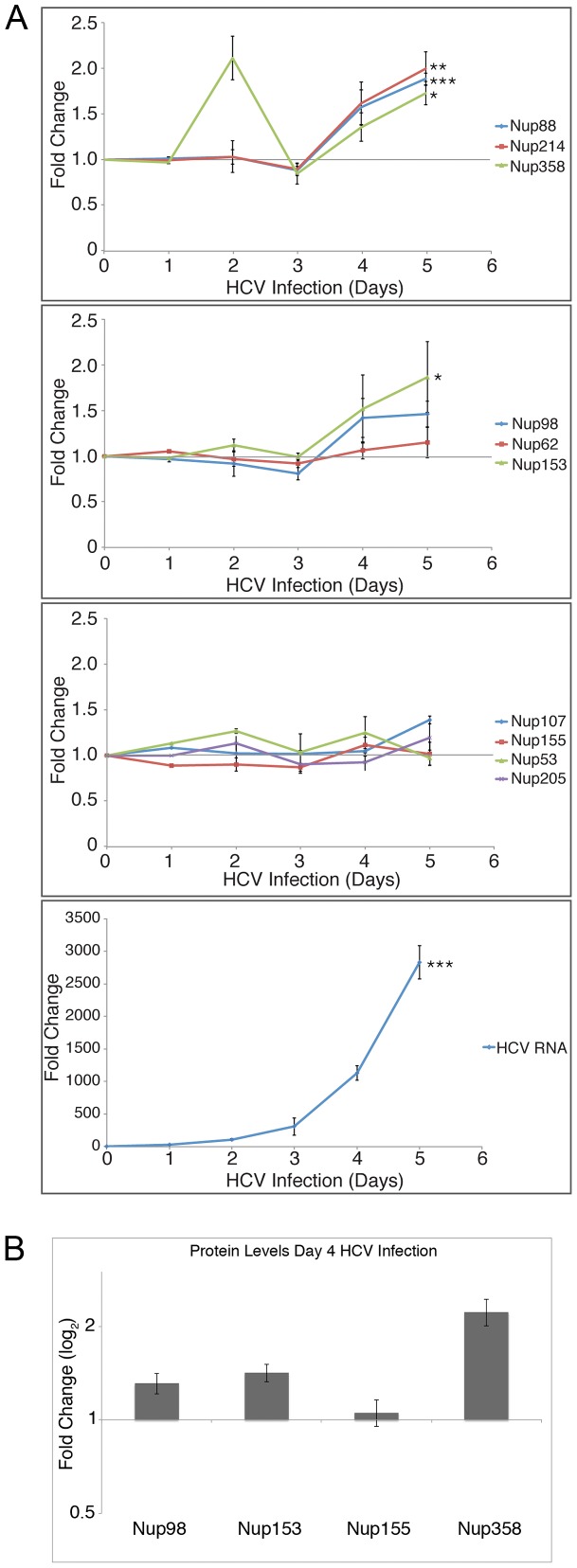
HCV infection increases RNA and protein levels of a subset of Nups. A) Total RNA was isolated from cell lysates at the indicated time points after infection of Huh7.5 cells with HCV, and levels of specific mRNA transcripts were assessed by qPCR. Values for each sample were normalized to HPRT and are expressed relative to uninfected cells (day 0 time point). HCV RNA levels are shown as fold change relative to HPRT. Error bars indicate standard error (based on ≥3 experiments) and statistical significance was evaluated using t-tests comparing each infected sample to an uninfected control sample. p-values less than 0.05 (*), 0.01 (**), and 0.001 (***) are indicated. B) Cell lysates were harvested from HCV infected Huh7.5 cells four days after infection and Nup proteins levels were determined by western blotting using antibodies specific for Nup98, Nup153, Nup155, and Nup358. Protein levels were quantified and normalized to tubulin levels and the fold-change is relative to uninfected cells. Error bars were determined using data from ≥3 experiments.

The accumulation of Nups in the vicinity of HCV assembly sites could arise from redistribution of cellular pools, increased cellular levels of these proteins, or a combination of both events. To assess the potential contribution of increased Nup synthesis, we examined cellular levels of various Nup mRNA transcripts at time points after HCV infection of Huh7.5 cells (Figure 3A). We found that mRNA levels of Nups composing the cytoplasmic filaments of the NPC (Nup88, Nup214 and Nup358), and one that is part of the nuclear basket (Nup153) were reproducibly elevated 1.5- to 2-fold four days after HCV infection (Figure 3A) in a manner that qualitatively paralleled increasing HCV RNA levels. In addition, Nup358 showed a reproducible biphasic pattern with an additional peak visible at 2 days after infection. By contrast, levels of transcripts encoding for several Nups that make up the scaffold of the NPC (including Nup155, Nup107, Nup53, and Nup205) and two associated Nups, Nup62 and Nup98, showed little or no change during HCV infection. Consistent with the changes in transcript levels, quantitative analysis of a subset of these Nups by western blotting showed increased levels of Nup98, Nup153, and Nup358, but not Nup155 (Figure 3B). These results indicate that a subset of Nups are up-regulated during HCV infection while others show no statistically significant change. Thus, the Nups recruited to sites of viral assembly are likely to arise from both constitutive and HCV-induced Nup expression.

### HCV core and NS5A interact with Kap β3 and Kap α

**Figure 4:**
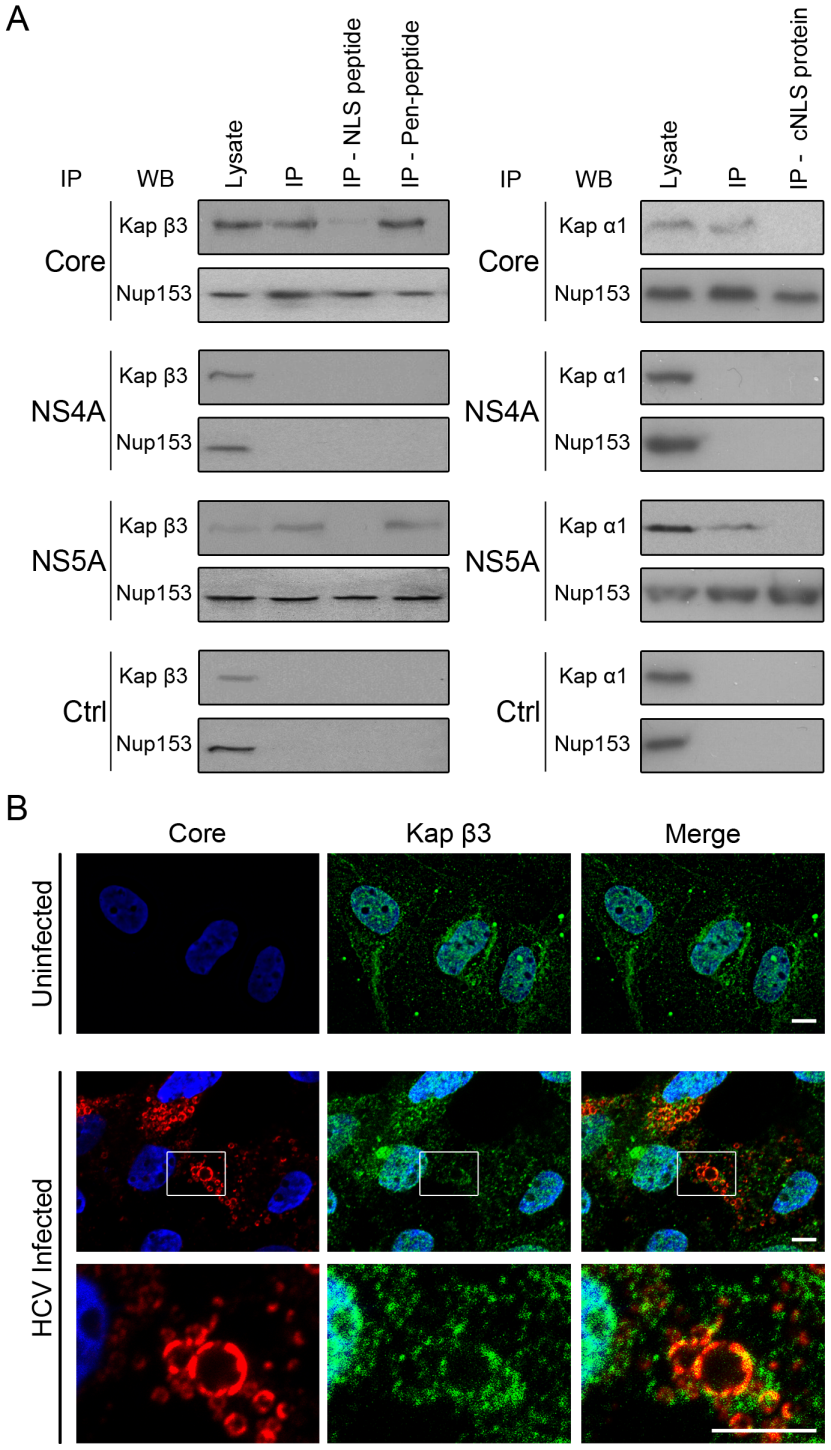
Identification of Kaps that physically interact with HCV proteins through NLS sequences. A) Constructs encoding the indicated V5-tagged HCV protein or an empty V5 vector (Ctrl) were transfected into HEK293T cells. 12 hrs after transfection, cells were subjected to no treatment, treatment with Kap β3/IPO5-NLS peptides, treatment with control penetratin peptides, or transfection with a construct encoding for a cNLS-GFP fusion protein and incubated for an additional 36 hrs. Following treatment, cells were lysed and V5-tagged HCV proteins were immunoprecipitated with anti-V5 antibodies (IP). Associated proteins were detected by western blotting using antibodies against the indicated Nup or Kap (WB). B) The subcellular localization of Kap β3/IPO5 in Huh7.5 cells uninfected or infected with HCV was determined by indirect immunofluorescence microscopy using antibodies specific to Kap β3/IPO5 (green) and HCV core (red). DNA was stained with DAPI (blue). Scale bars, 5 µm.

To further understand the physical and functional basis for the interaction of HCV proteins with Nups, we considered the potential role of putative NLS sequences (i.e. potential nuclear transport factor binding domains) present in several HCV proteins, including core and NS5A. NLS sequences can bind Kaps, which, in turn, could mediate the interactions of the HCV proteins with Nups. Therefore, we tested whether these HCV proteins were capable of binding Kaps and whether this interaction was responsible for their binding to Nups. The NLS sequences in Core and NS5A are predicted to bind specific members of the Kap family: Kap β3/IPO5 and the Kap α/β1 complex ([18], [19] unpublished data). Thus, we immunoprecipitated V5-tagged core, NS5A, or NS4A from cell extracts and probed for associated Kap β3/IPO5 and the Kap α/β1 complex. Antibodies directed against Kap β3/IPO5 and Kap α detected these proteins in association with core and NS5A but not NS4A, which lacks a predicted NLS (Figure 4A). These interactions could be inhibited by competing NLS-containing peptides. Treatment of cells with cell-penetrating Kap β3/IPO5-specific NLS peptides or the overexpression of a Kap α-specific NLS (cNLS)-containing reporter protein blocked the interactions of core and NS5A with these Kaps. However, disruption of Kap interactions with core and NS5A did not alter their binding to Nup153, Nup107, or Nup155, showing that binding of these HCV proteins to Nups is not mediated by Kaps (Figure 4A and S8A).

Considering the redistribution of Nups observed in HCV infected cells, we examined whether the cellular distribution of Kap β3/IPO5 was also altered in these cells. In uninfected cells, immunofluorescence microscopy analysis detected Kap β3/IPO5 both within the nucleus and the cytoplasm. In HCV infected cells, a similar distribution was observed, however Kap β3/IPO5 appeared enriched in regions of the cytoplasm adjacent to, or occupied by, HCV core protein (Figure 4B). This recruitment of Kap β3/IPO5 to centers of HCV assembly does not appear to arise from increased expression of the Kap β3/IPO5 encoding gene, as cellular levels Kap β3/IPO5 mRNA were not significantly increased in HCV infected cells (Figure S8B). Similarly, levels of Kap β1 and Kap α mRNAs were also not significantly changed.

### Nups and Kap β3/IPO5 support HCV infection

**Figure 5:**
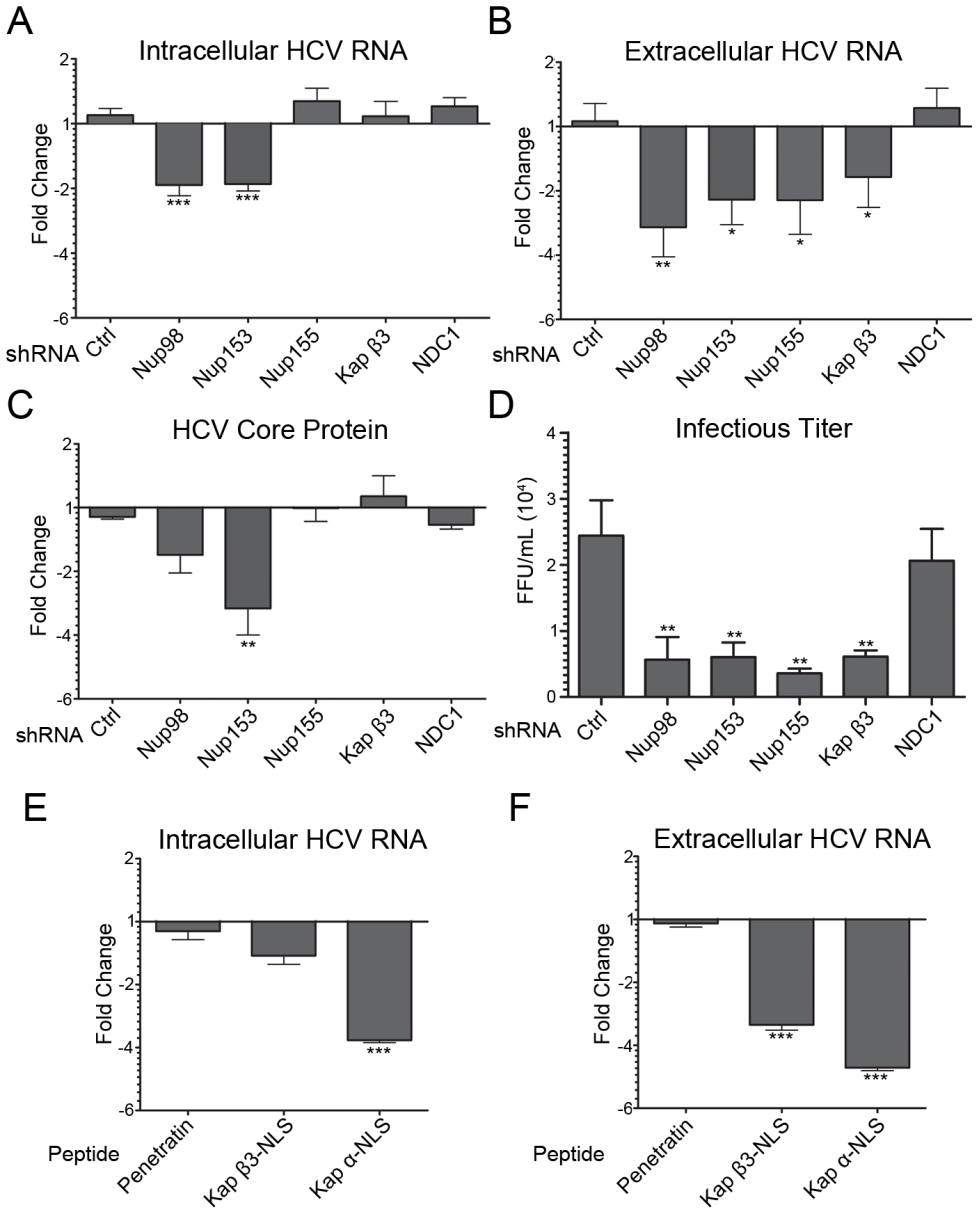
Depletion of Nups and Kaps inhibits HCV replication. A–D) Huh7.5 cells were coinfected with HCV and lentivirus encoding shRNAs directed against Nup98, Nup155, Nup153, Kap β3/IPO5, NDC1, or a scrambled control sequence for four days. The effects of Nup or Kap depletion on HCV titers were evaluated by qPCR analysis of HCV RNA levels in cell extracts (panel A) or in the culture supernatant (panel B) from HCV infected Huh7.5 cells co-infected with and without lentivirus. In addition, intracellular levels of the HCV core protein were examined by quantitative western blotting using antibodies specific for HCV core (panel C). Values for each sample are normalized to HPRT mRNA (Panel A and B) or α-tubulin (Panel C) and are expressed as fold-change relative to HCV infected cells not treated with lentivirus. Error bars indicate standard error (based on ≥3 experiments) and statistics are based on t-tests comparing each Nup or Kap specific shRNA treated sample to samples expressing the scrambled shRNA control. D) Huh7.5 cells were grown as described in panel A and the infectious titers of HCV particles present in the media of cells depleted of the indicated proteins were determined. Focus-forming units were determined using indirect immunofluorescence microscopy. Values shown represent focus-forming units per mL of medium (FFU/mL). E–F) Huh7.5 cells were infected with HCV and 4 hrs post infection a penetratin peptide, a Kap β3-NLS peptide, or a penetratin peptide containing a N-terminal Kap α NLS (Kap α-NLS) was added to the media. Four days later the effects of these peptides on HCV RNA levels in intracellular (panel E) and extracellular (panel F) compartments were assessed by qPCR analysis. Values for each sample are normalized to HPRT mRNA levels and expressed as fold change relative to cells receiving no peptide. Error bars indicate standard error (based on ≥3 experiments) and statistics based on t-tests comparing cells treated with penetratin alone to those treated with the Kap β3-NLS peptide or the Kap α-NLS containing peptide. p-values less than 0.05 (*), 0.01 (**), and 0.001 (***) are indicated.

To examine the relevance of interactions between HCV proteins and Nups or Kaps, we investigated the consequences of reducing cellular levels of specific Nups and Kap β3/IPO5 on HCV replication. Lentivirus expressing shRNAs were used to reduce levels of targeted proteins. Using this approach, mRNA and protein levels for each of the targeted genes were decreased by >60% in Huh7.5 cells by 4 days after lentivirus transduction (Figure S9A and S9B) with little effect on cell viability (Figure S9C). Cells were coinfected with lentivirus and HCV and, 4 days post infection, intracellular and extracellular HCV RNA levels were determined using quantitative real-time PCR (qPCR)(Figure 5A, 5B, and S9E). The results of these experiments revealed that intracellular levels of viral RNA were significantly decreased upon depletion of Nup98 or Nup153 (Figure 5A and S9E), while reduced levels of Nup155, NDC1, or Kap β3/IPO5, or treatment with lentivirus encoding a scrambled control sequence, had no effect. Consistent with these observations, quantitative western blotting revealed a decrease in HCV core protein levels in Nup98- or Nup153-depleted cells (Figure 5C). In accordance with decreased intracellular viral RNA levels, cells depleted of Nup98 or Nup153 also showed similar decreases in the levels of secreted virus (Figure 5B). Although Nup155- or Kap β3/IPO5-depleted cells showed no change in intracellular levels of HCV RNA, extracellular levels of secreted virus were decreased in these cells (Figure 5B), suggesting a requirement for Nup155 and Kap β3/IPO5 at a post-replication stage of virus assembly or in viral egress. These divergent effects of Nup depletions on intracellular versus extracellular RNA levels suggest functions for Nups at different stages of the HCV infectious cycle.

On the basis of our results, we concluded that at least a subset of Nups and Kap β3/IPO5 function in the production of secreted HCV. To further evaluate the relationship between the extracellular HCV RNA and the state of extracellular virus in the Nup and Kap depleted cells, cells were co-infected with HCV and lentivirus, and the infectious titer of virus in the medium was determined (Figure 5D). Consistent with our results showing decreases in extracellular HCV RNA levels, we also observed decreases in HCV infectious titers following depletion of Nup98, Nup153, Nup155 and Kap β3/IPO5 (Figure 5D). When normalized to the amount of released HCV RNA, we observed that, with the exception of Nup155 depleted cells showing a slight decrease in the specific infectivity of viral particles, none of the knockdown cells produced viral particles with a significantly lower specific infectivity than virus from control cells (Figure S9D). Thus, we concluded that depletion of specific Nups decreases the efficiency of HCV replication and/or assembly but did not change viral particle infectivity.

Our observation that depletion of Kap β3/IPO5 reduces levels of secreted virus led us to further examine the role of Kaps in HCV infection by inhibiting interactions with NLS-containing targets *in vivo* using synthetic NLS-containing peptides [23], [35]. As discussed in the previous section, these peptides can disrupt interaction of HCV proteins with Kaps in cells, however, they do not significantly affect cell viability (Figure S9F). As shown in Figure 5E and 5F, treatment of HCV infected cells with the Kap α-NLS peptides significantly decreased both intracellular and extracellular HCV RNA levels. In comparison, Kap β3/IPO5-NLS peptides resulted in only a slight decrease in intracellular HCV RNA levels, but a significant decrease in the levels of secreted virus (Figure 5E and 5F). These data are similar to that obtained upon depletion of Kap β3/IPO5 (Figure 5A, 5B, and 5D). Combined with the decrease in HCV titers observed upon depletion of Kap β3/IPO5 (Figure 5B and 5D), the decrease in HCV titers following treatment of infected cells with NLS peptides provides further evidence that the nuclear transport pathways are important for viral infection and that, like Nups, different Kaps may contribute to distinct stages in the viral life cycle.

### Nuclear transport substrates accumulate in regions of HCV assembly

**Figure 6:**
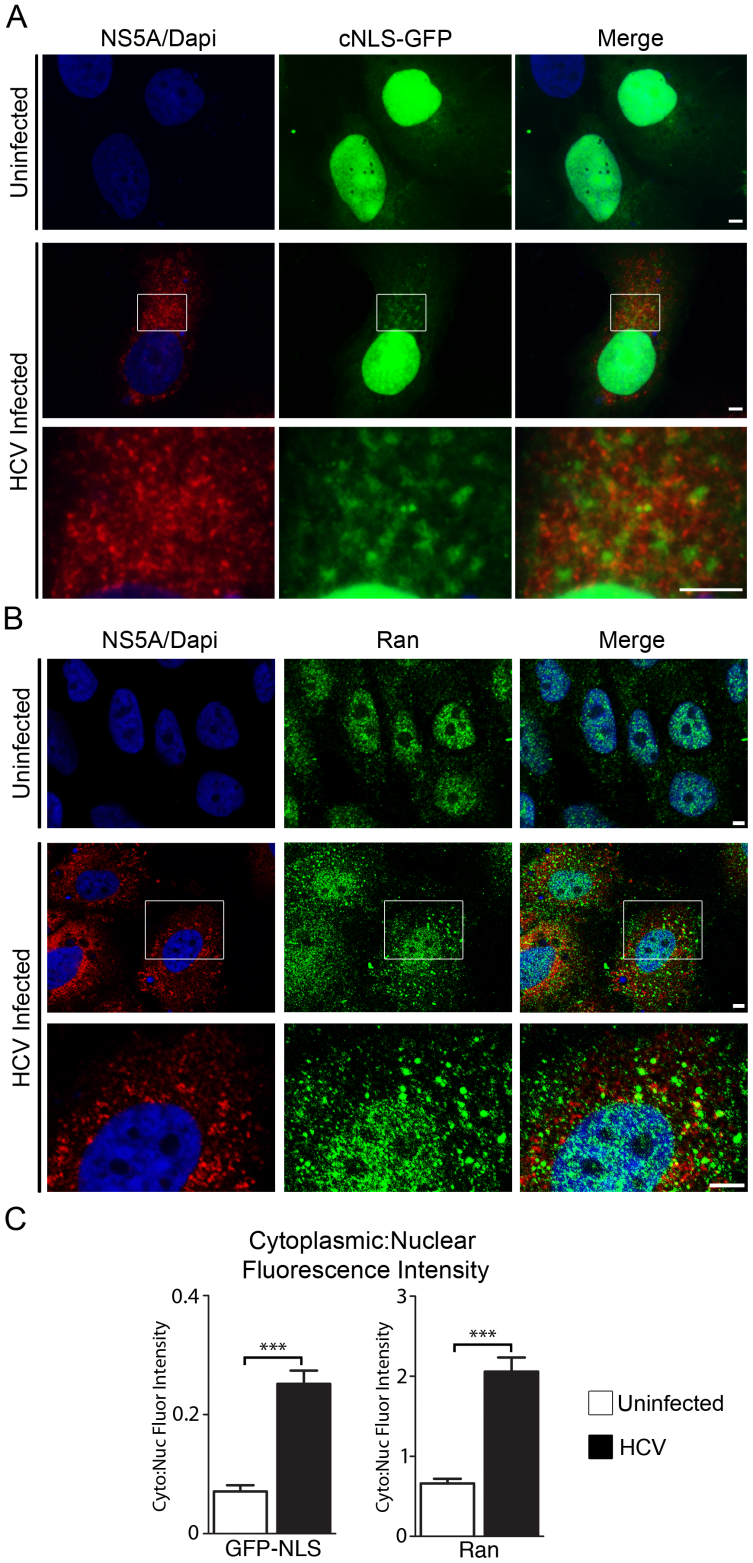
cNLS-GFP reporter protein and Ran accumulated in the vicinity of HCV replication/assembly. A) Uninfected or HCV-infected Huh7.5 cells were transfected with a construct encoding a chimeric protein consisting of an N-terminal cNLS sequence followed by two tandemly repeated GFP molecules 2 days after infection. On day 4 after HCV infection, the cNLS-GFP reporter was visualized by fluorescence microscopy (green) and its location compared to HCV NS5A (red) detected by immunofluorescence microscopy. DNA was detected with DAPI (blue). All cell producing the cNLS-GFP reporter exhibited a nuclear signal. Visible levels of the cNLS-GFP reporter were also detected in the cytoplasm of ∼81% of infected cells (n = 193) and ∼23% of uninfected cells (n = 198). Scale bars, 5 µm. B) Localization of Ran in Huh7.5 cells, either uninfected or infected with HCV for four days, was evaluated by indirect immunofluorescence microscopy using antibodies specific for Ran (green) and HCV NS5A (red). DNA was stained with DAPI (blue). Scale bar, 5 µm. C) Cytoplasmic and nuclear fluorescence signal levels produced by the cNLS-GFP reporter protein or the Ran specific antibodies were used to determine cytoplasmic to nuclear fluorescence ratios in uninfected and HCV infected cells. Fluorescence levels were calculated using ImageJ software and the statistical significance of differences in the ratios detected in uninfected versus HCV-infected cells was evaluated using t-tests. Asterisks (***) denotes a p-value of less than 0.001.

The HCV-induced membranous web is thought to restrict access of cytoplasmic factors, such as pattern recognition receptors, from sites of HCV replication and assembly. The accumulation of Nups and Kap β3/IPO5 in the vicinity of HCV replication and assembly sites led us to investigate the potential role of the nuclear transport machinery in mediating access of molecules to compartments within the membranous web. We hypothesized that, like their role at the NE, the presences of nuclear transport factors within the membranous web could facilitate access of NLS-containing molecules, including NLS-containing HCV proteins such as core and NS5A, into regions within the membranous web through their interactions with Kaps. Therefore, we examined whether NLS-containing proteins could access regions of the membranous web in HCV-infected Huh7.5 cells using a chimeric gene encoding two tandemly repeated GFP proteins fused to a canonical SV40 NLS (cNLS). The cNLS-GFP fusion protein accumulated efficiently in the nuclei of uninfected cells, with little or no signal visible in the cytoplasm or associated with cytoplasmic membrane structures (Figure 6A). HCV infected cells also exhibited a robust nuclear accumulation of the cNLS-GFP fusion protein suggesting nuclear import was functional in these cells. However, in the HCV infected cells significantly higher levels of cytoplasmic signal were detected (Figure 6C). Importantly, the cNLS-GFP fusion protein was not diffusely distributed throughout the cytoplasm, but rather it appeared in distinct regions of the cytoplasm that were adjacent to or occupied by core and NS5A (Figure 6A and S10).

The concentration of the cNLS reporter in regions of the cytoplasm occupied by the membranous web are consistent with a role for Kaps and NPCs in regulating access to compartments within the membranous web. A key factor responsible for the accumulation of cargoes in the nucleoplasm is the small GTPase Ran, which, when bound to GTP, binds import Kaps and induces release of attached cargoes. Therefore, we examined whether in HCV infected cells Ran was present in the cytoplasm in addition to it normal concentration in the nucleoplasm. In uninfected Huh7.5 cells, Ran was detected primarily to the nucleus, consistent with previous studies [36]. However, in HCV infected cells, we detected a clear change in the localization pattern of Ran. While still present in the nucleus, infected cells contained cytoplasmic pools of Ran largely concentrated in multiple foci (Figure 6B). Quantification of the fluorescent intensity in cytoplasmic and nuclear compartments of uninfected and HCV infected cells confirmed the increase in cytoplasmic Ran levels (Figure 6C). This cytoplasmic localization of Ran further supports the conclusion that the nuclear transport machinery functions in the cytoplasmic compartment to support HCV replication/assembly.

## Discussion

**Figure 7:**
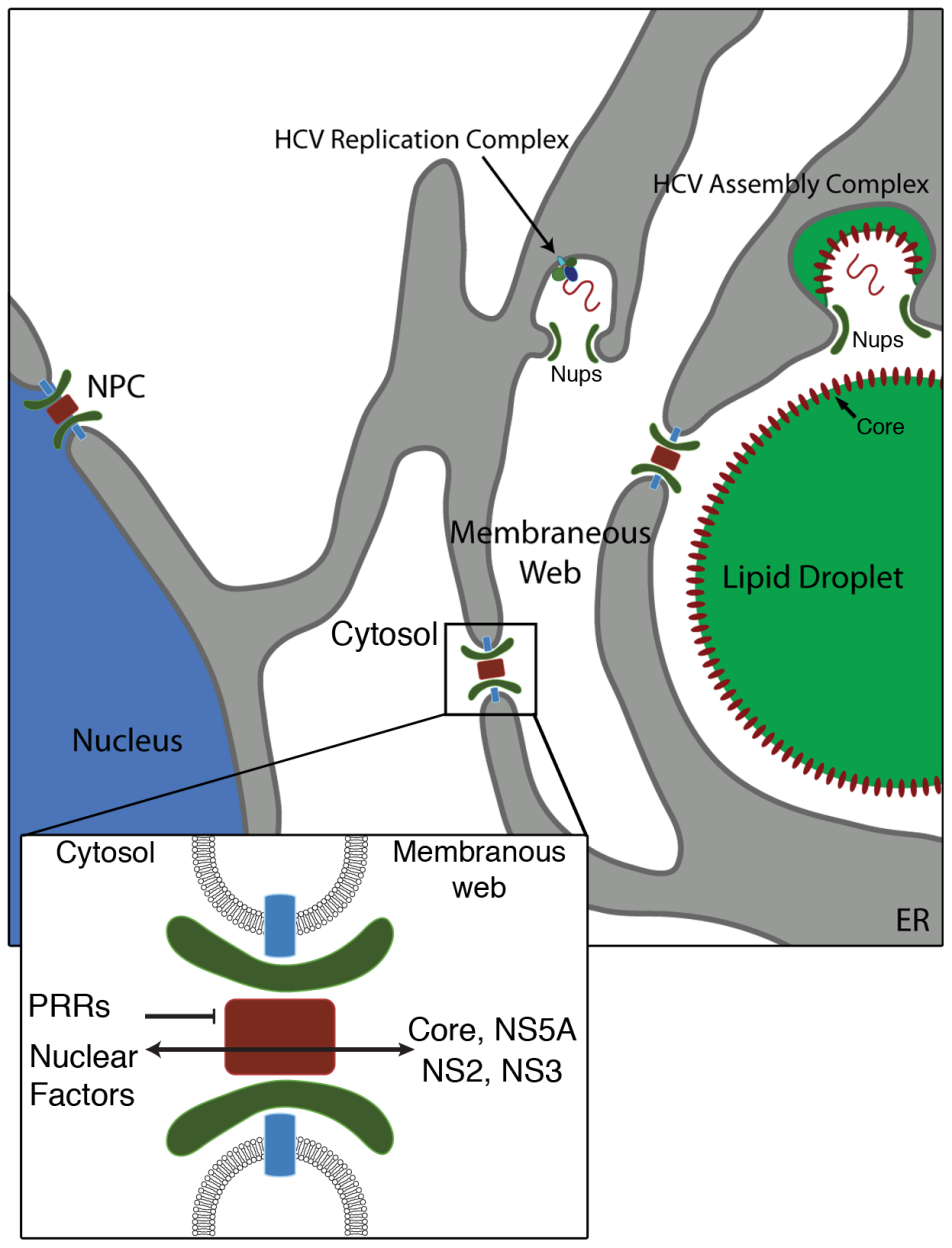
Model for the function of cytoplasmic NPCs in HCV infection. In HCV infected cells, cytoplasmically positioned NPCs are predicted to form channels across double membrane structures of the membranous web. These NPCs are proposed to facilitate movement of NLS-containing proteins, such as HCV core, NS2, NS3, NS5A and host nuclear proteins, from the surrounding cytoplasm across double membrane structures of the membranous web while excluding proteins lacking NLS sequences, such as pattern recognition receptors (PRRs), from regions of HCV replication and assembly events. We also speculate that Nups (such as Nup155 and Nup153) may be recruited to regions of the membranous web where HCV replication and virion assembly would employ the ability of these Nups to contour membranes.

The organization, composition, and functions of membrane structures induced by positive strand RNA viruses remain largely ill-defined. During HCV infection, it has been postulated that the membranous web functions in viral egress, concentration and synchronization of viral replication and assembly, as well as avoidance from host cytoplasmic PRRs [5], [13]. All of these functions are thought to require the existence of a permeability barrier between the cytosol and the interior of the membranous web. Here we report that Nups accumulate in the membranous web at sites of HCV replication or assembly. Consistent with these observations, we detect various HCV proteins in association with specific Nups and Kaps. Importantly, these proteins play a role in HCV infection. Insight into the function of these interactions came from the observation that a reporter protein normally found exclusively in the nucleus is also targeted to regions of the cytoplasm occupied by the membranous web. We hypothesize that Nups and Kaps present in the virally-induced membranous web facilitate virus replication, in part, through their ability to sequester molecules, both host and HCV proteins, required for HCV replication and assembly (Figure 7).

The existence of assembled cytoplasmic NPCs crossing ER membranes, or annulate lamellae, has been reported in many cell types [29], [30]. These NPCs are capable of transporting NLS-containing cargo across the ER [37], but the function they play in the cytoplasm is unclear. We have observed that, during HCV infection, multiple Nups are redistributed to cytoplasmic membranes enriched with HCV proteins (Figure 1, S2, S3, S4, and S6). Indeed, HCV infection resulted in the re-localization of all the Nups examined, representing most of the major subcomplexes of the NPC, to regions of the cytoplasm populated by HCV proteins. These results lead us to conclude that intact cytoplasmic NPCs, or derivatives of these structures, are present in areas of HCV replication or assembly. This concept is consistent with previous electron microscopy studies of HCV infected cells where various double membrane structures, topologically analogous to ER-like structures housing cytoplasmic NPCs, were detected in the membranous web [9], [38], [39].

Changes in the localization of Nups in HCV infected cells led us to investigate corresponding changes in mRNA transcript and protein levels. Our qPCR analysis of Nup and Kap transcript levels revealed that only a subset are elevated in HCV infected cells, indicating that Nups recruited to the membranous web are likely derived from both existing cellular Nup pools and increased synthesis. Several Nups have previously been observed to be up-regulated upon innate immune stimulation of specific cell types [40], [41]. Similar innate immune activation has also been observed upon HCV infection leading to the possibility that the observed up-regulation of Nups in HCV infected cells may result from HCV-mediated immune activation [42], [43]. However, this does not appear to be the case as treatment of Huh7.5 cells with various immune stimulants did not alter cellular levels of mRNAs encoding many of the Nups examined in this study (Figure S11). Thus, the increase in Nup levels observed following HCV infection likely occurs through a mechanism distinct from immune activation.

Immunoprecipitation experiments revealed that several Nups associate with HCV core and NS5A. Additionally, the HCV channel forming protein, p7, has previously been shown to interact with Nup214 in a yeast two-hybrid system [44]. Importantly, the interactions between Nups and HCV core or NS5A are not mediated by Kaps (Figure 4A and S8A). Thus, their interactions are unlikely to reflect a transport intermediate where HCV proteins are moving as cargo through the NPC. Rather, these data are consistent with a direct association between HCV proteins and Nups. These interactions are predicted to contribute to the recruitment of specific Nups, as well as associated subcomplexes or assembled NPCs, to the forming membranous web.

It is possible that the interaction of HCV proteins with Nups and kaps could potentially alter host cell nucleocytoplasmic transport in such a way that facilitates HCV replication. For example some viruses, including polio and influenza, inhibit nuclear transport by inducing Nup degradation, to improve viral replication [22], [23], [24]. However, it seems less likely that HCV proteins target Nups and Kaps for a similar reason as we do not detect degradation of Nups, and nuclear import of the cNLS-GFP reporter, while exhibiting some cytoplasmic localization, appears robust in HCV-infected cells (Figure 1, 6, and S10). Instead, we hypothesize that interactions of HCV protein and Nups and Kaps reflect two, potentially simultaneously acting, functions conducted by these proteins within the membranous web. One proposed role is based on the observation that a subset of Nups, including Nup107 and Nup155, are structurally related to secretory vesicle coat proteins. These proteins have highly conserved domain structures and their interactions with membranes and membrane proteins is proposed to facilitate the convex membrane curvature of the pore membrane domains that connect the inner and outer nuclear membranes and attach to the scaffold structures of the NPC [45]. In addition, while lacking structural similarity to coat proteins, Nup153 interacts with the vesicle coat protein COPI and this association has been linked to the post-mitotic NE membrane assembly [46]. The association of Nup153 with HCV proteins and COPI is also intriguing in light of studies showing a role for the COPI coatomer complex in HCV replication [47]. Considering these observations, we speculate that Nups may be recruited to the membranous web, in part, to usurp their functions in contouring of membranes. Importantly, the curvature of membrane domains at sites of viral particle budding into the ER lumen is topologically similar to the pore membrane [48], [49]. These ideas are consistent with the physical association of Nup155 and Nup153 with core and NS5A and the visible close association of these Nups with core enriched regions adjacent to lipid droplets (Figure 1).

Our data also support a second, more conventional role for Nups within the membranous web as part of assembled or partially assembled NPCs and as regulators of Kap/cargo movement. We envisage that the membranous web-associated NPCs selectively allow NLS-containing molecules to access regions within the membranous web. Such a mechanism would explain several observations. For example, previous studies have identified, or inferred the presence of, NLS-like sequences or NTF binding domains in HCV proteins, including in core [17], NS5A [15], and NS3 [14], and our own analysis has also revealed NLS-like sequences in these proteins and NS2 (unpublished data). Moreover, previous studies have detected interactions between HCV proteins and Kaps [18], [19], [20], and we have found that core and NS5A proteins interact with Kap β3/IPO5 and Kap α (Figure 4). However, since these proteins are ER-associated and detected in the cytoplasm during infection, we propose that these import signals function in the cytoplasm.

A requirement for a transport regulatory mechanism within the membranous web is inferred by the observed compartmentalization properties of this structure, including several studies showing HCV RNA and proteins within the membranous web are resistant to RNase and protease treatment [12]. This physical separation is also revealed by the exclusion of tubulin from regions of the cytoplasm occupied by HCV proteins (Figure S1B and S1C). Various HCV and host proteins synthesized in the cytosol must overcome this barrier to enter regions of the web where HCV replication and assembly occurs. We propose that NLS sequences within HCV proteins as well as several host-cell nuclear factors detected in the membranous web [50], [51] function to facilitate movement of these proteins from the cytosol through NPCs positioned in the membranous web to regions of HCV replication and assembly. Importantly, cytoplasmic proteins lacking NLSs, such as PRRs, would be inhibited from accessing viral RNA; events potentially contributing to the ability of HCV to maintain a chronic infection. This concept of NPC-mediated transport functioning within the membranous web is directly supported by our observations that a cNLS-GFP reporter protein is visibly enriched in regions of the cytoplasm occupied by HCV proteins (Figure 6 and S10). Furthermore, the concentration of Kap β3/IPO5 in regions of the cytoplasm occupied by core (Figure 4) supports a function for this NTF in the membranous web.

Consistent with these proposed functions for the nuclear transport machinery within the membranous web, we observe that various Nups and Kaps are required for HCV production. For example, we detected an inhibition of HCV replication or assembly following depletion of Nup98, Nup153, or Nup155 (Figure 5). Depleted levels of mRNA and protein for each Nup appeared similar (Figure S9), however, the consequences of their depletion on HCV replication were not. While depletion of Nup98 or Nup153 reduced both intracellular levels of viral RNA and secreted virus, depletion of Nup155 led to a specific decrease in secreted virus but no significant change in intracellular levels of viral RNA. These results are consistent with Nup98 and Nup153 being required prior to or coincident with in HCV RNA replication. By contrast, Nup155 depleted cells show no defect in intracellular HCV RNA accumulation implying that Nup155 contributes to post-replication processes such as effective viral packaging or egress. These differential effects of Nup depletion remain to be further characterized and we envisage several potential scenarios that would explain these results. They may arise from the different functional roles of these Nups within NPCs, thus depleting individual Nups would lead to distinct changes in the functionality of the NPC, including alterations in the functions of specific transport pathways. In support of this idea, inhibition of Kap α transport by treatment with competitive peptides mirrors the effects of depleting Nup98 or Nup153, namely decreasing both intracellular HCV RNA and secreted virus. Conversely, depletion of Kap β3/IPO5, or competitive inhibition of its *in vivo* function with peptides, results in a phenotype similar to depletion of Nup155, namely a decrease in secreted virus but no change in the intracellular levels of HCV RNA (Figure 5). Thus, distinct transport pathways may have functions at different stages of the HCV lifecycle, likely as defined by their cargos. Alternatively, the structural integrity and general transport functions of the NPC appear to be differentially tolerant to changes in the levels of individual Nups. This is revealed, for example, by depletion of NDC1, where NPCs remain functional despite significant depletion [52] of what is thought to be an essential component of the NPC [53], [54]. The presence of depleted, but functional, NPCs would explain our observation that depletion of NDC1 did not significantly alter HCV replication. Another possibility is that these Nups and Kaps also contribute to virus production through additional functions unlinked to transport. For example, as discussed above, Nups such as Nup155 likely influence membrane structure; moreover, various Nups, including Nup98 and Nup155 have been linked to the maintenance of chromatin structure and the regulation of transcription (reviewed in [45], [55]).

Our model suggesting that Nups form functional NPCs within the membranous web implies NPCs are capable of functioning outside the confines of the nuclear envelope. Indeed previous studies have shown that annulate lamellae can transport NLS-coupled gold particles across the ER [37]. Moreover, a recent report demonstrated that NPCs are present at the transition zone of cilia in mammalian cells and that a transport mechanism similar to that of nucleocytoplasmic transport is utilized to transport proteins between the cilia and the adjacent cytoplasm [56], [57]. Interestingly, these cilia associated NPCs appear to lack certain Nups suggesting they represent derivatives of the NE embedded structures. Whether the NPCs we have detected associated with the membranous web also represent a variant form of NPCs remains to be determined. This seems plausible as our replicon data imply that HCV proteins do not recruit intact NPCs to the membranous web but rather distinct HCV proteins may recruit different subsets of Nups. We can envisage that the concerted activities of the HCV proteins could promote assembly of NPCs or a variant form of this structure in the membranous web.

The ability of HCV to exploit the functions of the Nups and Kaps for the purpose of creating an environment conducive to its replication and assembly may represent a mechanism widely used by positive-strand RNA viruses. For example, we have observed increased amounts of cytoplasmic Nup98-containing foci that likely represent NPCs in cells infected with hepatitis A virus and dengue virus (Figure S5). Consistent with this observation, electron microscopy studies have reported increased levels of annulate lamellae in hepatitis A virus infected cells [58], as well as cells infected with Japanese Encephalitis virus and Rubella virus [59], [60], [61]. These results lead us to conclude that Nups represent a conserved target of positive-strand RNA viruses and, with greater mechanistic understanding, a potential target for antiviral intervention.

## Materials and Methods

### Cell culture, viral infection and immune stimulation

HEK293T, A549, Huh7.5, and Huh7 cells were maintained in DMEM (Sigma) containing 10% FBS (Sigma). For HCV infection, Huh7.5 cells were seeded at a density of 2.5×10^5^ cells/well in 6-well tissue culture plates, and 24 hrs after plating, they were infected with 3 RNA genome equivalents of a serially passaged JFH-1 strain of HCV. For HAV infection, Huh7 cells were grown to 70% confluence and infected with HAV/p16 virus at and MOI of 0.1. Cells were analyzed by immunofluorescence 3 weeks after infection. For dengue virus infection, A549 cells were grown to a density of 2.5×10^5^ cells/well in 12-well tissue culture plates, and infected with DENV-2 strain of dengue virus at an MOI of 1. Dengue virus infected cells were examined by immunofluorescence 48 hrs after infection. Huh7 cells containing the JFH-1 strain subgenomic replicon (encoding NS3 through to the C-terminus of the HCV polyprotein) were maintained in DMEM containing 10% FBS and 500 mg/mL G418. For immune stimulation experiments Huh7.5 cells were incubated over a time course of 24 hrs with human recombinant interferon alpha (1000 units/mL)(Intron A, DIN02238675), human recombinant interferon gamma (500 units/mL)(PBL, 11500-1), human recombinant TNF-α (100 ng/mL)(Sigma, T152) or γ-irradiated LPS (100 ng/mL)(Sigma, L7770).

### Quantitative real-time PCR (qPCR)

Total RNA was extracted from cells using Trizol (Invitrogen, 15596018) or from supernatants using a High Pure Viral Nucleic Acid Kit (Roche, 11858874001). Relative mRNA transcript levels were determined by real-time PCR using either SYBR-green or TaqMan master mixes. For details, see extended Materials and Methods.

### Immunofluorescence and western blotting

Immunofluorescence and western blotting were done as previously described [53]. Further details and a list of primary and secondary antibodies are provided in the extended Materials and Methods. Quantification of nuclear and cytoplasmic fluorescence levels was done using ImageJ. Cell outlines were determined using DIC images. The percent cytoplasmic colocalization of Nups with HCV core or the NS5A protein was calculated in ImageJ using the Manders overlap coefficients as previously described [62]. Pearson's colocalization coefficients were calculated as previously described using the JACoP plugin for ImageJ [63].

### Subcellular fractionation

Subcellular fractionation was performed as previously described [31], [33]. For details, see extended Materials and Methods.

### Expression constructs and transfection

Expression constructs for production of HCV proteins were made using sequences from the H77 strain. DNA sequences encoding core (amino-acid residues 1–191), NS4A (residues 1638–1711) and NS5A (residues 1973–2416) were cloned into a pcDNA3.1/nV5-DEST expression vector (Invitrogen, 12290010) using the gateway cloning system (Invitrogen). The cNLS-GFP construct has been previously described [64]. Constructs were transfected into HEK293T or Huh7.5 cells using lipofectamine 2000 reagent (Invitrogen, 11668019) and expressed for 48 hrs.

### Immunoprecipitation

HEK293T cells were transfected with pcDNA3.1/nV5-DEST plasmid alone or encoding HCV core, NS4A, or NS5A and proteins were immunoprecipitated using anti-V5 antibodies. Interactions were evaluated by western blotting using antibodies described in the extended Materials and Methods.

### Synthetic peptides

Synthetic peptides including Kap α-NLS-penetratin, Kap β3/IPO5-NLS [23], [35], and penetratin [65] have been previously described. For details, see extended Materials and Methods.

### Production of shRNA-expressing lentiviral particles

Lentivirus particles were produced using previously described viral packaging vectors [66] and pLKO.1 vectors containing the various shRNA sequences (Sigma) listed in Table S2. Lentiviral titers were determined by infecting HEK293T cells with serially diluted lentiviral stocks and selecting for transduced cells with Puromycin. For lentiviral transduction, 2.5×10^5^ Huh7.5 cells were plated in 6-well tissue culture plates and infected with lentivirus at an MOI of 2. Four days after infection, cells were harvested and transcript or protein levels were evaluated using qPCR or western blotting.

### MTT cell viability assay

A MTT (3-(4,5-dimethylthiazol-2-yl)-2,5-diphenyltetrazolium bromide) cell viability assay was used to determine cytotoxicity of shRNA expression or peptide treatment [67], [68].

### Specific infectivity and infectious titer assays

HCV particles were harvested from the culture media of cells coinfected with HCV and lentivirus. Media was then diluted to make viral stocks containing 1×10^5^ HCV RNA copies/mL. These viral stocks were serially diluted and added to Huh7.5 cells grown in optical 96 well plates. 2 days after infection, viral focus-forming units were determined by indirect immunofluorescence microscopy using antibodies specific to HCV core protein. The values for specific infectivity were calculated by dividing the number of Focus forming units by the total number of HCV RNA copies added to the cells (FFU/HCV RNA copy). The values for infectious titer represent the number of focus forming units per mL in the culture medium harvested from each of the coinfected samples. The values for specific infectivity and infectious titer show an average over a count of 6 wells, and each experiment was repeated 3 times.

### Accession numbers

Nup53- NM_138285, Nup62- NM_012346, Nup88- NM_002532, Nup98- NM_016320, Nup107- NM_020401, Nup153- NM_005124, Nup155- NM_153485, Nup205- NM_015135, Nup214- NM_005085, Nup358- NM_006267, NDC1- NM_001168551, Lamin B- NM_032737, Kap β3/IPO5- NM_002271, Kap α1- NM_002264, Kap α6- NM_012316, Imp β1- NM_002265, IFNα- V00548, IFNγ- V00543, TNFα- NM_000594, β Tubulin- NM_006000, VAC- NM_003374.2, FACL4- AB061713.1, HPRT- NM_000194.2, HCV JFH- HM049503, and HCV H77- JX472013.

## Supporting Information

Figure S1 **Localization of core and NS5A proteins in HCV infected Huh7.5 cells.** Huh7.5 cells were infected with HCV for four days. A) The subcellular localization of lipid droplets and HCV core was determined by fluorescence confocal microscopy using BODIPY (green) and antibodies directed against HCV core (red). Boxed areas in the top rows of images are show at higher magnification in the bottom row. DNA was stained with DAPI (blue). B and C) The subcellular localization of tubulin (green) and HCV core (panel B, red) or NS5A (panel C, red) was examined by indirect immunofluorescence confocal microscopy using antibodies directed against the indicated proteins. DNA was stained with DAPI (blue). Scale bars, 5 µm.
(TIF)

Figure S2 **Localization of Nups and lamin B in HCV infected cells.** **A**) Localization of Nups and the nuclear lamina was evaluated in uninfected Huh7.5 cells (Un) or 4 days following infection with HCV (HCV) by indirect immunofluorescence microscopy using antibodies specific for the indicated Nups or lamin B (green). HCV core protein localization was determined using anti-core antibodies (red). DNA is stained with DAPI (blue). These images provide lower magnification views of similar test samples examined in Figure 1. Scale bars, 5 µm. B) Percent colocalization between cytoplasmic Nups and HCV core protein in an average of ≥10 cells processed as in panel A was determined. Values represent the percent of the cytoplasmic Nup fluorescence signal overlapping with the HCV core fluorescence signal calculated using the Manders colocalization coefficient.
(TIF)

Figure S3 **Colocalization between Nups and HCV core proteins.** Huh7.5 cells were infected with HCV for 4 days. The localization of Nups and lamin B compared to HCV core was evaluated using indirect immunofluorescence by staining with antibodies specific for Nups and lamin B (green) or HCV core (red). Fluorescence intensity line graphs were plotted using pixel intensity data obtained from red and green fluorescence channels along lines (white) drawn through regions containing core protein and lipid droplets. DNA was stained with DAPI (blue). Scale bars, 2 µm.
(TIF)

Figure S4 **Localization of Nups and tubulin in HCV infected cells.** A–B) The localization of Nup98 or Nup155 and tubulin was evaluated in uninfected or HCV infected Huh7.5 cells four days after infection. Cells were examined by indirect immunofluorescence confocal microscopy using antibodies directed against Nup155 (panel A, green) or Nup98 (panel B, green) and tubulin (red). DNA was stained with DAPI (blue). Scale bars, 5 µm.
(TIF)

Figure S5 **Cytoplasmic localization of Nups in dengue and hepatitis A virus infected cells.** A) The localization of Nup98 was examined by indirect immunofluorescence microscopy in uninfected Huh7 cells and cells infected with hepatitis A virus (HAV infected) for three weeks using anti-Nup98 antibodies (green). Hepatitis A viral RNA was detected using anti-dsRNA antibodies (red). B) Localization of Nup98 was also examined in dengue virus infected A549 cells (2 days post infection) as described in panel A (green) and compared to the localization of dengue virus capsid protein using capsid specific antibodies (red). In both panels, DNA was stained with DAPI (blue). Scale bars, 5 µm.
(TIF)

Figure S6 **Localization of Nups and HCV proteins in transfected or HCV-infected cells.** A) Huh7.5 cells were transfected with constructs encoding for V5-tagged HCV core, NS5A, or NS4A. The localization of the Nup98 and tagged HCV proteins was examined 48 hours later, by indirect immunofluorescence confocal microscopy using anti-Nup98 (green) and anti-V5 (red) antibodies. Arrows point to cells expressing the indicated HCV protein and Pearson's colocalization coefficients are specified in the merge panel. DNA is stained with DAPI (blue). Scale bars, 10 µm. B) The localization of Nup155 and Nup98 was evaluated in uninfected Huh7.5 cells (Un) or 4 days following infection with HCV (HCV) by indirect immunofluorescence confocal microscopy using antibodies specific for the indicated Nups (green) and HCV NS5A protein (red). Boxed areas in the middle rows of images are show at higher magnification in the bottom row. DNA is stained with DAPI (blue). Scale bars, 5 µm. C) Percent colocalization between cytoplasmic Nups and NS5A in an average of ≥10 cells processed as in panel B was determined. Values represent the percent of the cytoplasmic Nup fluorescence signal overlapping with the HCV core fluorescence signal calculated using the Manders colocalization coefficient.
(TIF)

Figure S7 **Localization of a subset of Nups in Huh7 cells expressing the JFH-1 subgenomic replicon.** Huh7 cells expressing or not expressing the JFH-1 subgenomic replicon (encoding NS3 through to the C-terminus of the HCV polyprotein) were analyzed by indirect immunofluorescence using antibodies directed against the HCV protein NS5A (red) and the indicated Nups (green). DNA is stained with DAPI (blue). Scale bars, 5 µm.
(TIF)

Figure S8 **NLS peptides and NLS-containing proteins do not disrupt interactions between HCV proteins and Nups.** A) Constructs encoding for the indicated V5-tagged HCV proteins were transfected into HEK293T cells and expressed for 48 hrs. Twelve hours after transfection, cells were subjected to no treatment, treatment with Kap β3/IPO5-NLS peptides, or treatment with control penetratin peptides. Alternatively, cells were transfected with a construct encoding for a cNLS-GFP fusion protein 12 hrs after transfection with HCV protein encoding constructs. Cells were then lysed and V5-tagged proteins were immunoprecipitated with anti-V5 antibodies (IP). Associated proteins were detected by western blotting using antibodies against the indicated proteins (WB). B) Huh7.5 cells were infected with HCV and total RNA was isolated from cell lysates at the indicated time points after infection. The mRNA transcript levels of the indicated Kaps were measured by qPCR analysis over a time course of infection. Values for each sample are normalized to HPRT mRNA and are expressed relative to mRNA levels of the indicated Kaps prior to infection (day 0 time point). Error bars indicate standard error (n = 3 experiments).
(TIF)

Figure S9 **shRNA-mediated knockdown of Nups and Kaps and their effects on cell survival and the specific infectivity of HCV.** A–D) Huh7.5 cells were coinfected with HCV and lentivirus encoding shRNAs directed against Nup98, Nup155, Nup153, NDC1, Kap β3, or a scrambled control sequence for four days. A) The level of mRNA transcript depletion following shRNA expression was evaluated by qPCR using primers specific for Nup98, Nup153, Nup155, Kap β3, or NDC1. Each sample is normalized to HPRT mRNA levels and expressed as a percent knockdown relative to cells infected with HCV and the lentivirus encoding the scrambled control shRNA sequence. B) Protein depletion following shRNA expression was evaluated by quantitative western blotting using antibodies specific for the indicated proteins. Protein levels were normalized to that of α-tubulin. Fold-change is relative to cells infected with HCV and the lentivirus encoding the scrambled control shRNA sequence. C) The cytotoxic effects of expressing the various shRNA constructs in Huh7.5 cells were evaluated using an MTT cell viability assay. Values for the survival of cells depleted of the indicated proteins represent fold change relative to cells not infected with lentivirus. D) The specific infectivity of HCV particles produced from cells coinfected with lentivirus was assessed by first determining total number of HCV RNA copies/mL in the culture supernatant for each sample. Huh7.5 cells were then infected with identical amounts of HCV RNA copies/mL harvested from each of the coinfected cells and focus-forming units were determined using indirect immunofluorescence microscopy. Values represent focus-forming units per copy of viral RNA. E) Huh7.5 cells were coinfected with HCV and lentivirus encoding a scrambled control sequence or an shRNA directed against a secondary site in either Nup155 or Nup153 distinct from that used in the analysis shown in A–D. Four days after infection, the levels of extracellular and intracellular HCV RNA were determined by qPCR. The values are presented as fold change relative to cell infected with HCV but not lentivirus. F) The cytotoxic effect of treating Huh7.5 cells with penetratin alone, Kap β3-NLS peptides, or penetratin peptides containing a N-terminal Kap α NLS sequence was determined using an MTT cell viability assay. Values for the survival of cells treated with peptides represent fold change relative to untreated cells.
(TIF)

Figure S10 **Localization of cNLS-GFP reporter protein to the membranous web.** Huh7.5 cells were infected with HCV. Two days following infection, cells were transfected with a plasmid-born construct encoding a cNLS-GFP reporter. Four days post infection, the subcellular localization of the cNLS-GFP protein was examined by fluorescence confocal microscopy (green). The localization of the cNLS-GFP fluorescence signal was compared to that of HCV core (red, panel A) or NS5A (red, panel B) by indirect immunofluorescence confocal microscopy using specific antibodies. In panel A, boxed areas in the second rows of images are shown at higher magnification in the third row. Regions enriched for both the cNLS-GFP reporter and HCV core are highlighted in third row with white ovals. In all panels, uninfected Huh7.5 cells producing the cNLS-GFP reporter are also shown. DNA is stained with DAPI (blue). Scale bars, 5 µm.
(TIF)

Figure S11 **Activation of innate immune pathways does not alter levels of various Nups in Huh7.5 cells.** Huh7.5 cells were treated with IFNγ (500 units/mL), TNF-α (100 ng/mL), LPS (0.1 µg/mL), or IFNα (1000 units/mL). At various time points transcript levels of the indicated Nups were evaluated by qPCR. Samples were normalized to HPRT transcript levels and fold change is relative to samples derived from untreated cells.
(TIF)

Table S1 **List of real time qPCR primers used in this study.**
(DOC)

Table S2 **List of the shRNA sequences used to deplete Nups or Kaps in this study.**
(DOC)

Text S1 **Supplementary** **Materials and Methods** **.**
(DOC)
