# Supplementary figures and images for: Hepatitis C Virus-Induced Cytoplasmic Organelles Use the Nuclear Transport Machinery to Establish an Environment Conducive to Virus Replication
Source: PLoS Pathog. 2013 Oct 31;9(10):e1003744. doi: 10.1371/journal.ppat.1003744 (PMC3814334; doi:10.1371/journal.ppat.1003744)

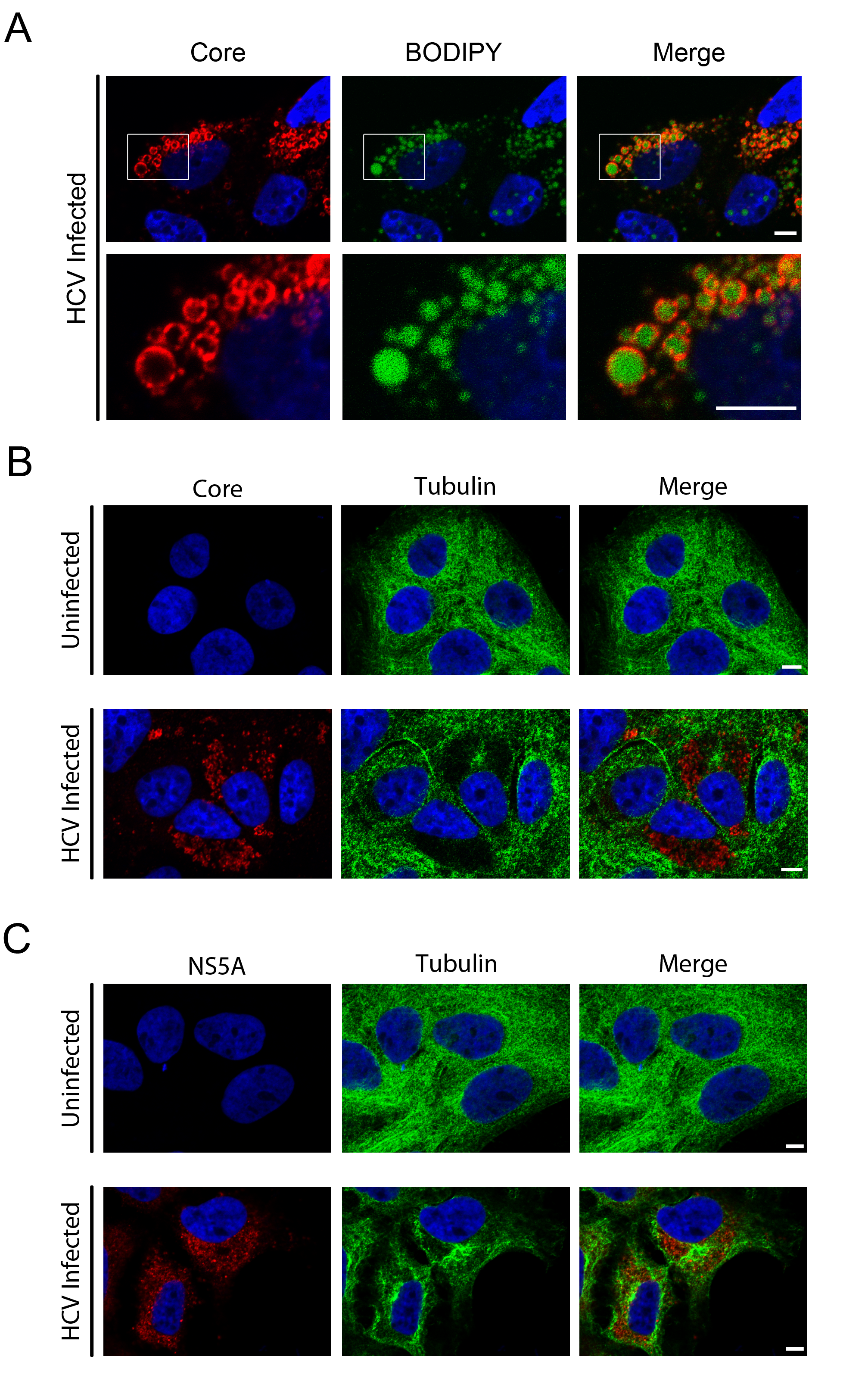

Supplement: Figure S1 — Localization of core and NS5A proteins in HCV infected Huh7.5 cells. Huh7.5 cells were infected with HCV for four days. A) The subcellular localization of lipid droplets and HCV core was determined by fluorescence confocal microscopy using BODIPY (green) and antibodies directed against HCV core (red). Boxed areas in the top rows of images are show at higher magnification in the bottom row. DNA was stained with DAPI (blue). B and C) The subcellular localization of tubulin (green) and HCV core (panel B, red) or NS5A (panel C, red) was examined by indirect immunofluorescence confocal microscopy using antibodies directed against the indicated proteins. DNA was stained with DAPI (blue). Scale bars, 5 µm. (TIF) [file ppat.1003744.s001.tif]

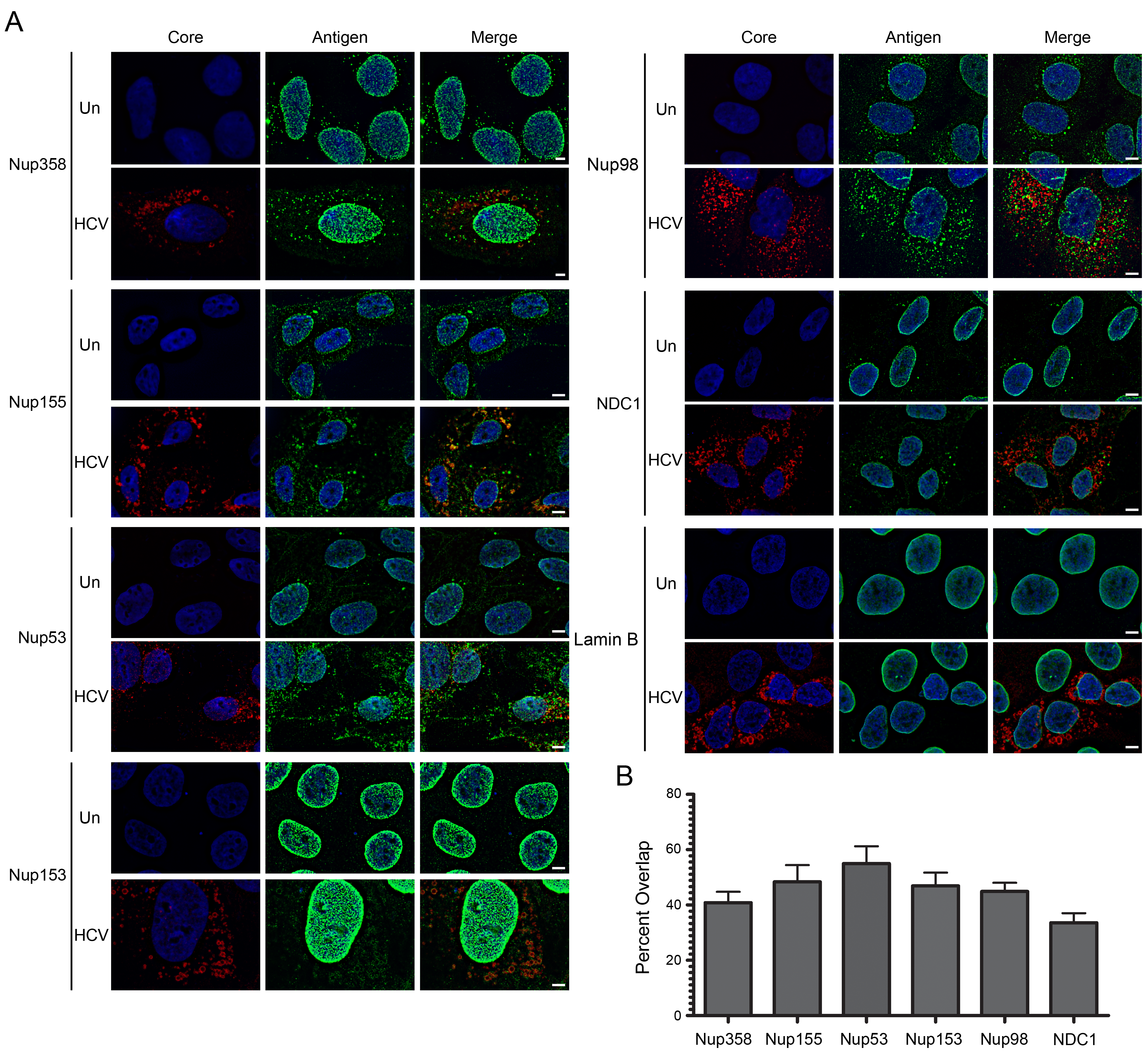

Supplement: Figure S2 — Localization of Nups and lamin B in HCV infected cells. A) Localization of Nups and the nuclear lamina was evaluated in uninfected Huh7.5 cells (Un) or 4 days following infection with HCV (HCV) by indirect immunofluorescence microscopy using antibodies specific for the indicated Nups or lamin B (green). HCV core protein localization was determined using anti-core antibodies (red). DNA is stained with DAPI (blue). These images provide lower magnification views of similar test samples examined in Figure 1. Scale bars, 5 µm. B) Percent colocalization between cytoplasmic Nups and HCV core protein in an average of ≥10 cells processed as in panel A was determined. Values represent the percent of the cytoplasmic Nup fluorescence signal overlapping with the HCV core fluorescence signal calculated using the Manders colocalization coefficient. (TIF) [file ppat.1003744.s002.tif]

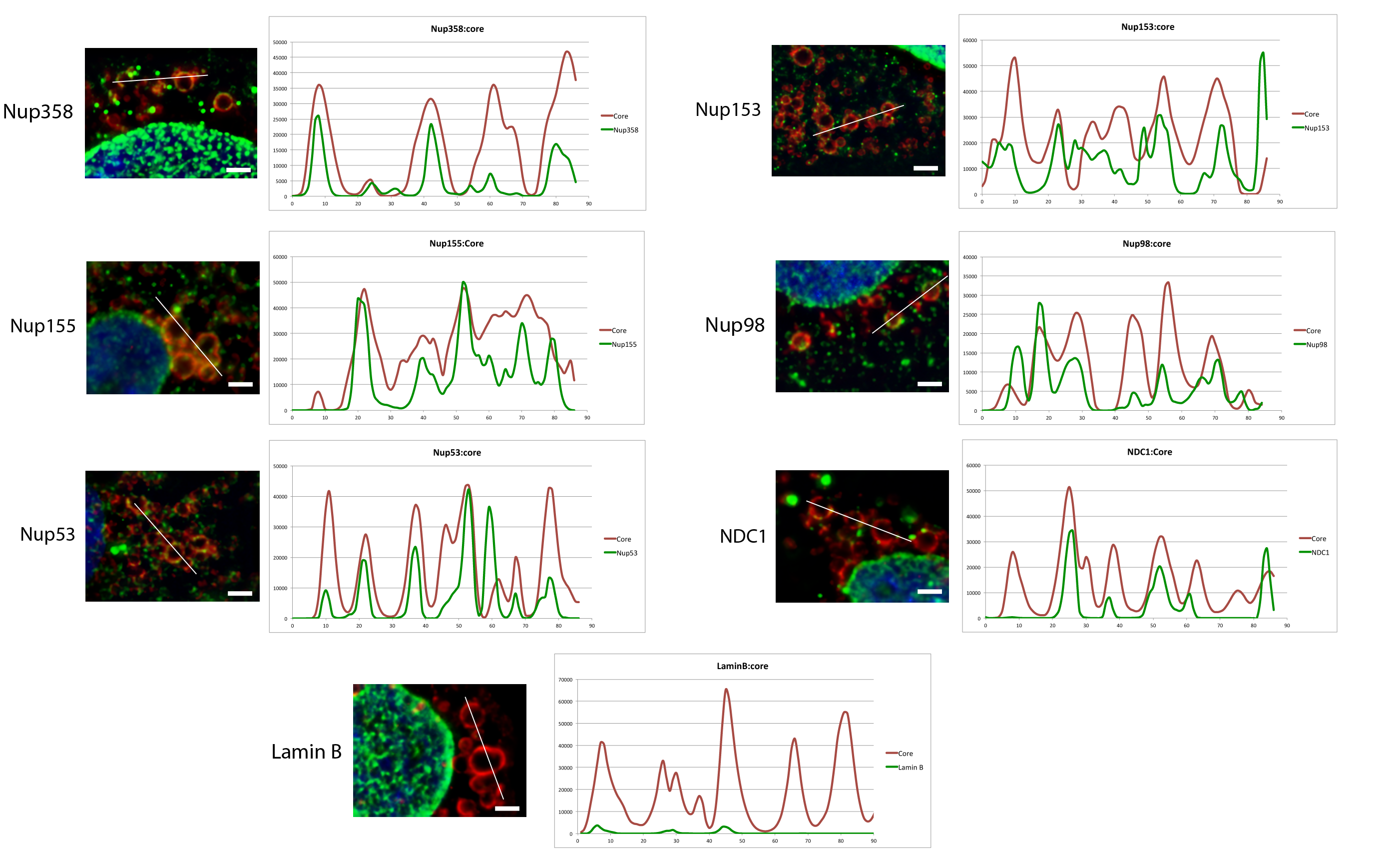

Supplement: Figure S3 — Colocalization between Nups and HCV core proteins. Huh7.5 cells were infected with HCV for 4 days. The localization of Nups and lamin B compared to HCV core was evaluated using indirect immunofluorescence by staining with antibodies specific for Nups and lamin B (green) or HCV core (red). Fluorescence intensity line graphs were plotted using pixel intensity data obtained from red and green fluorescence channels along lines (white) drawn through regions containing core protein and lipid droplets. DNA was stained with DAPI (blue). Scale bars, 2 µm. (TIF) [file ppat.1003744.s003.tif]

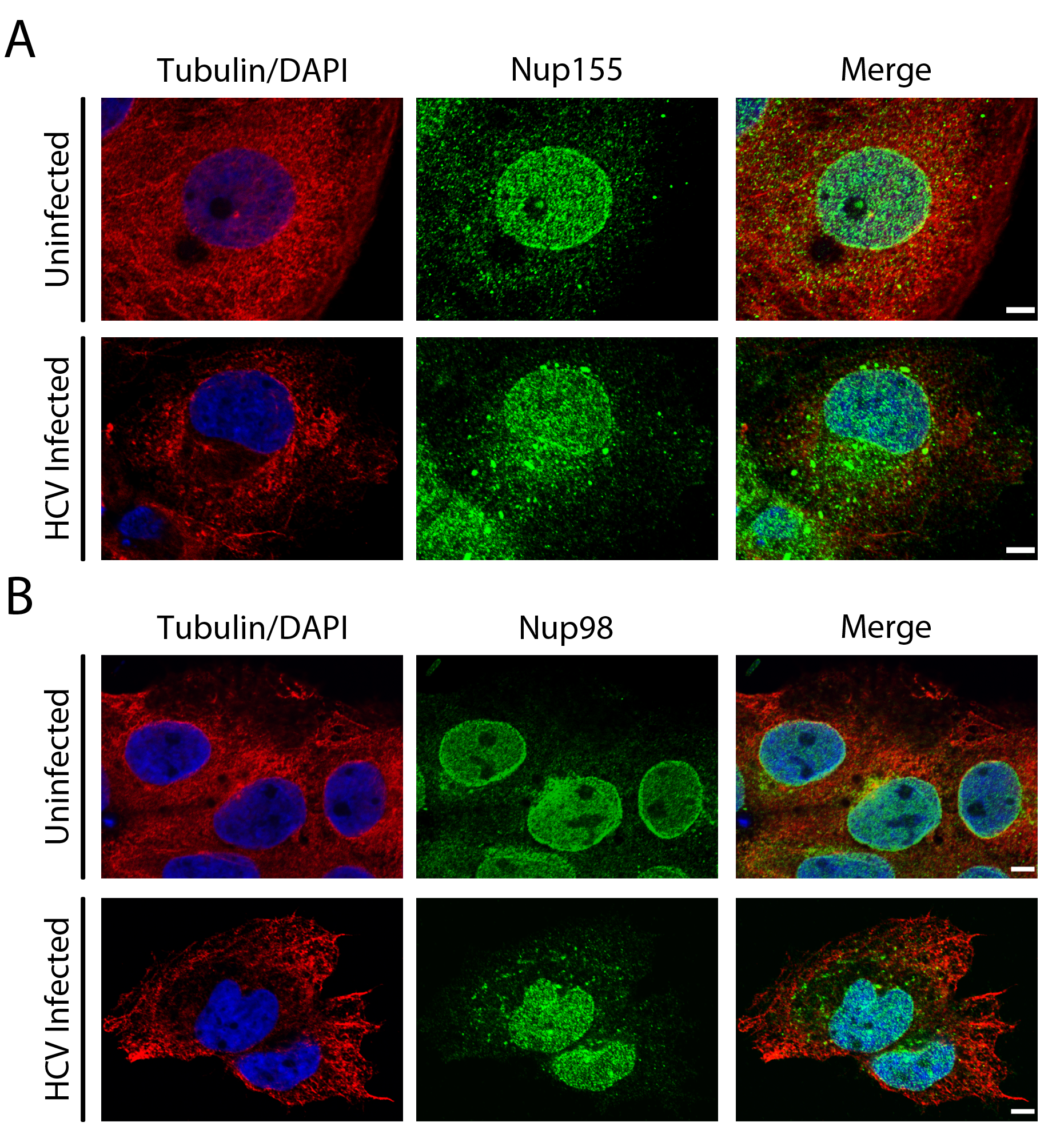

Supplement: Figure S4 — Localization of Nups and tubulin in HCV infected cells. A–B) The localization of Nup98 or Nup155 and tubulin was evaluated in uninfected or HCV infected Huh7.5 cells four days after infection. Cells were examined by indirect immunofluorescence confocal microscopy using antibodies directed against Nup155 (panel A, green) or Nup98 (panel B, green) and tubulin (red). DNA was stained with DAPI (blue). Scale bars, 5 µm. (TIF) [file ppat.1003744.s004.tif]

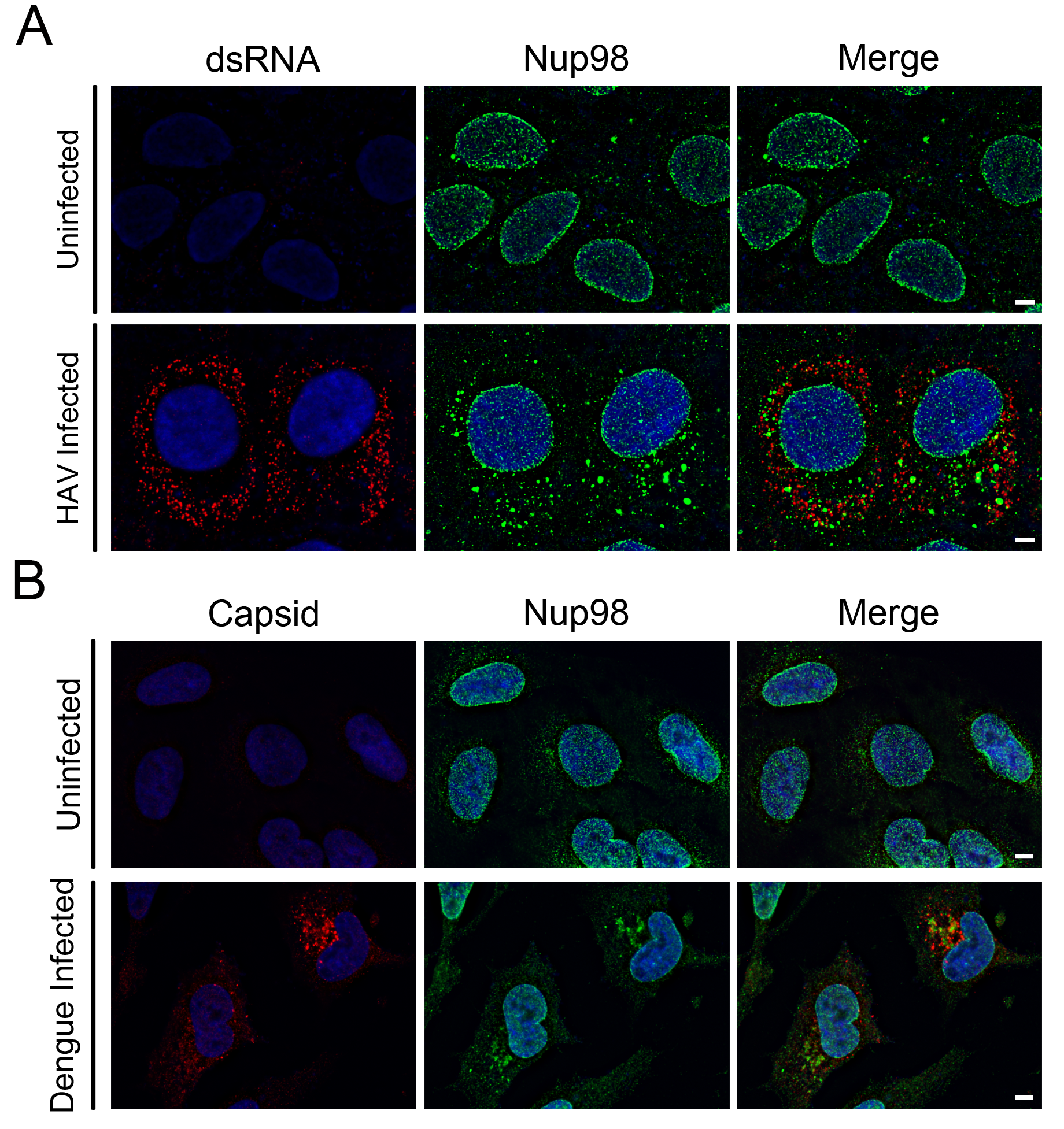

Supplement: Figure S5 — Cytoplasmic localization of Nups in dengue and hepatitis A virus infected cells. A) The localization of Nup98 was examined by indirect immunofluorescence microscopy in uninfected Huh7 cells and cells infected with hepatitis A virus (HAV infected) for three weeks using anti-Nup98 antibodies (green). Hepatitis A viral RNA was detected using anti-dsRNA antibodies (red). B) Localization of Nup98 was also examined in dengue virus infected A549 cells (2 days post infection) as described in panel A (green) and compared to the localization of dengue virus capsid protein using capsid specific antibodies (red). In both panels, DNA was stained with DAPI (blue). Scale bars, 5 µm. (TIF) [file ppat.1003744.s005.tif]

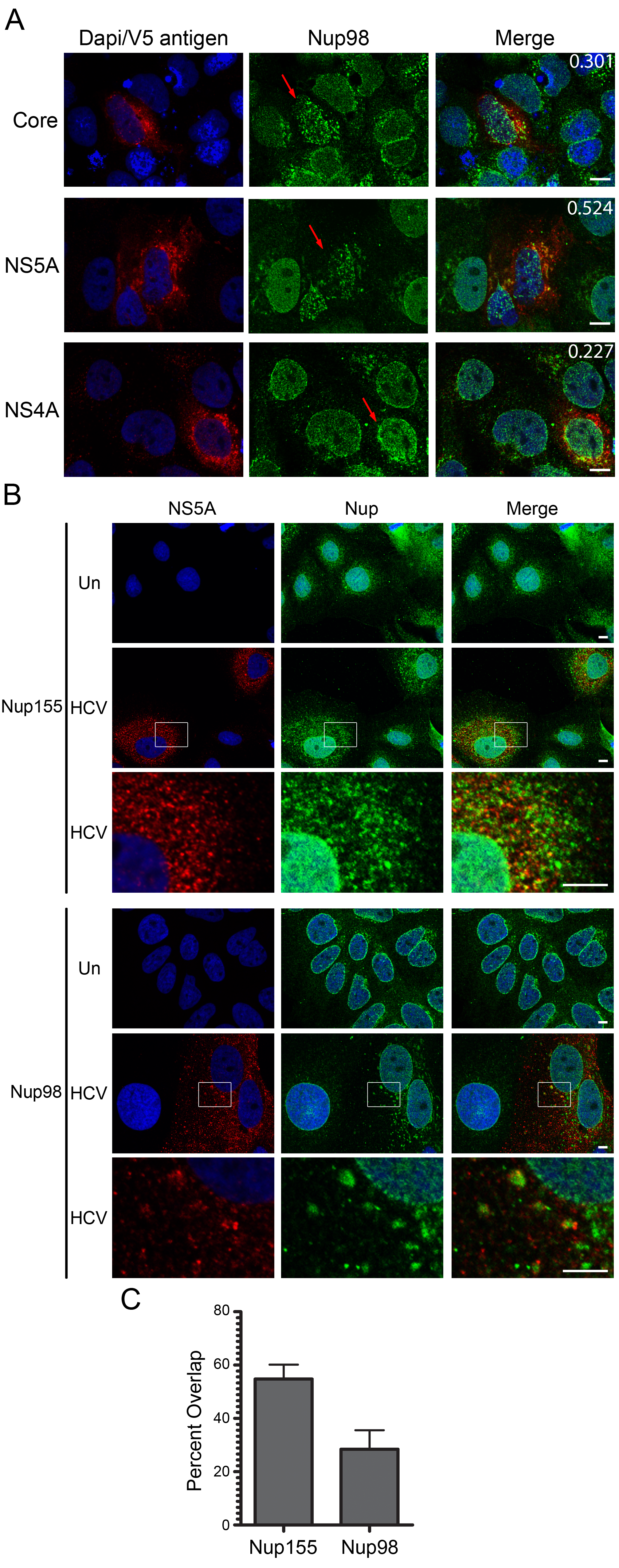

Supplement: Figure S6 — Localization of Nups and HCV proteins in transfected or HCV-infected cells. A) Huh7.5 cells were transfected with constructs encoding for V5-tagged HCV core, NS5A, or NS4A. The localization of the Nup98 and tagged HCV proteins was examined 48 hours later, by indirect immunofluorescence confocal microscopy using anti-Nup98 (green) and anti-V5 (red) antibodies. Arrows point to cells expressing the indicated HCV protein and Pearson's colocalization coefficients are specified in the merge panel. DNA is stained with DAPI (blue). Scale bars, 10 µm. B) The localization of Nup155 and Nup98 was evaluated in uninfected Huh7.5 cells (Un) or 4 days following infection with HCV (HCV) by indirect immunofluorescence confocal microscopy using antibodies specific for the indicated Nups (green) and HCV NS5A protein (red). Boxed areas in the middle rows of images are show at higher magnification in the bottom row. DNA is stained with DAPI (blue). Scale bars, 5 µm. C) Percent colocalization between cytoplasmic Nups and NS5A in an average of ≥10 cells processed as in panel B was determined. Values represent the percent of the cytoplasmic Nup fluorescence signal overlapping with the HCV core fluorescence signal calculated using the Manders colocalization coefficient. (TIF) [file ppat.1003744.s006.tif]

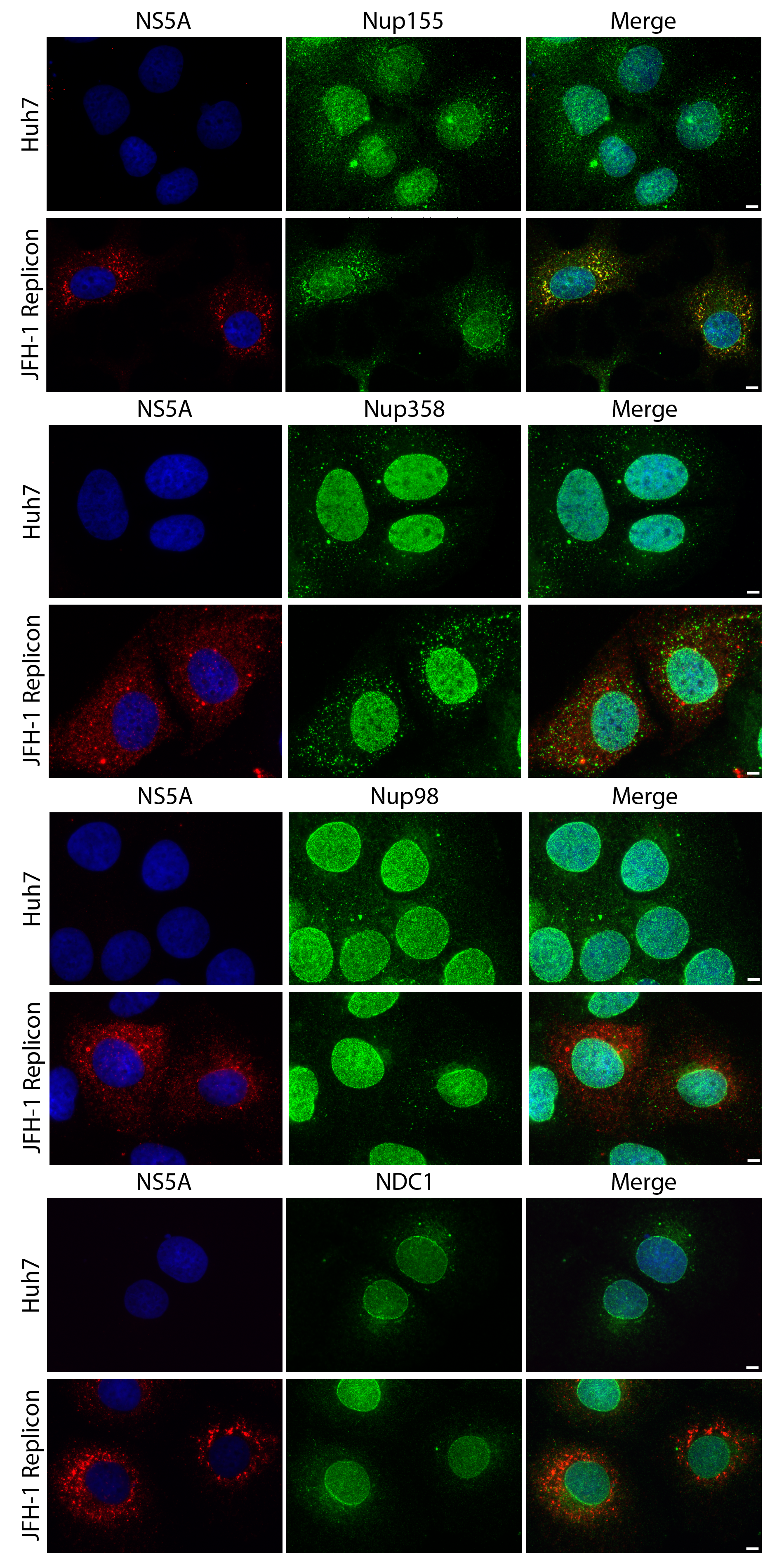

Supplement: Figure S7 — Localization of a subset of Nups in Huh7 cells expressing the JFH-1 subgenomic replicon. Huh7 cells expressing or not expressing the JFH-1 subgenomic replicon (encoding NS3 through to the C-terminus of the HCV polyprotein) were analyzed by indirect immunofluorescence using antibodies directed against the HCV protein NS5A (red) and the indicated Nups (green). DNA is stained with DAPI (blue). Scale bars, 5 µm. (TIF) [file ppat.1003744.s007.tif]

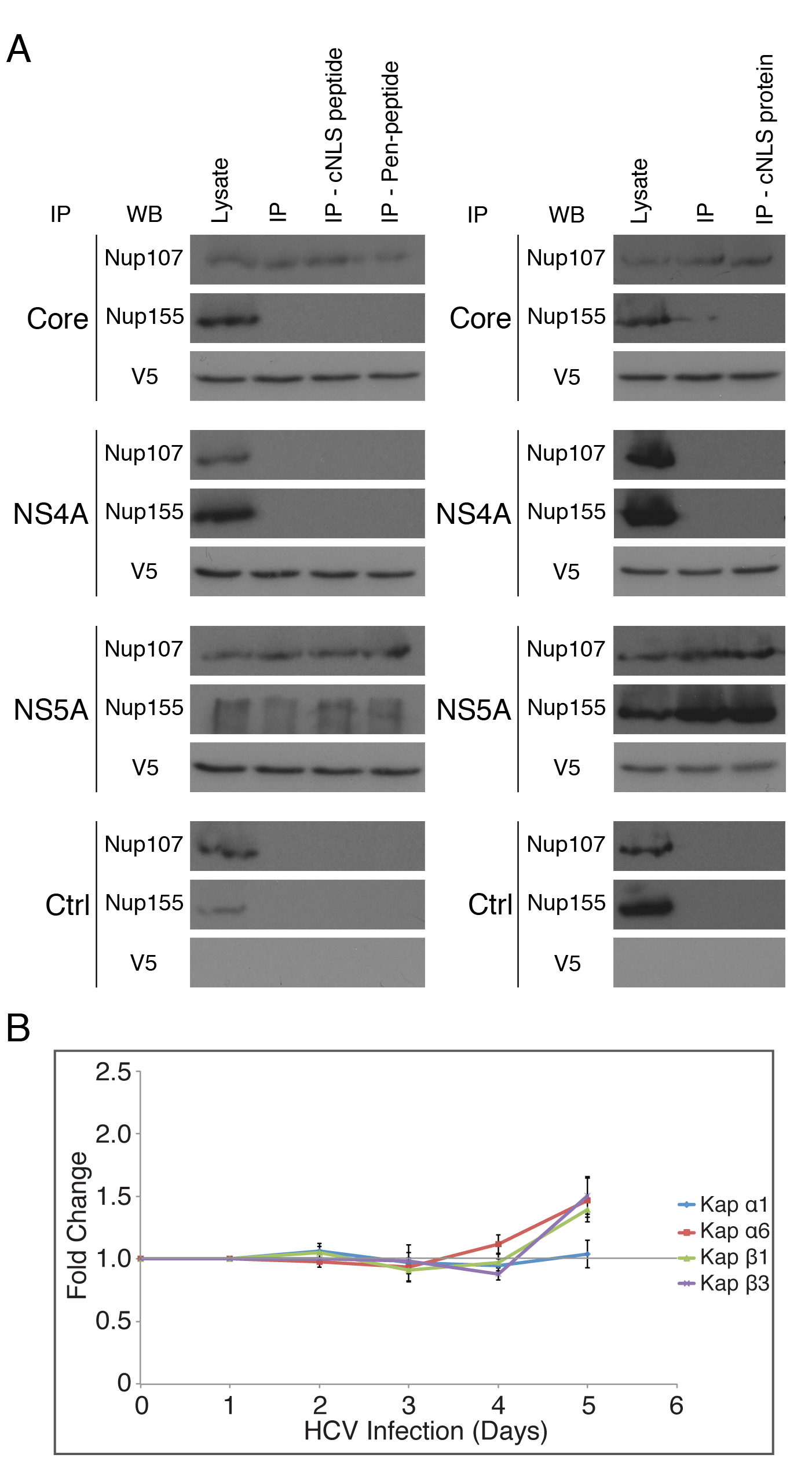

Supplement: Figure S8 — NLS peptides and NLS-containing proteins do not disrupt interactions between HCV proteins and Nups. A) Constructs encoding for the indicated V5-tagged HCV proteins were transfected into HEK293T cells and expressed for 48 hrs. Twelve hours after transfection, cells were subjected to no treatment, treatment with Kap β3/IPO5-NLS peptides, or treatment with control penetratin peptides. Alternatively, cells were transfected with a construct encoding for a cNLS-GFP fusion protein 12 hrs after transfection with HCV protein encoding constructs. Cells were then lysed and V5-tagged proteins were immunoprecipitated with anti-V5 antibodies (IP). Associated proteins were detected by western blotting using antibodies against the indicated proteins (WB). B) Huh7.5 cells were infected with HCV and total RNA was isolated from cell lysates at the indicated time points after infection. The mRNA transcript levels of the indicated Kaps were measured by qPCR analysis over a time course of infection. Values for each sample are normalized to HPRT mRNA and are expressed relative to mRNA levels of the indicated Kaps prior to infection (day 0 time point). Error bars indicate standard error (n = 3 experiments). (TIF) [file ppat.1003744.s008.tif]

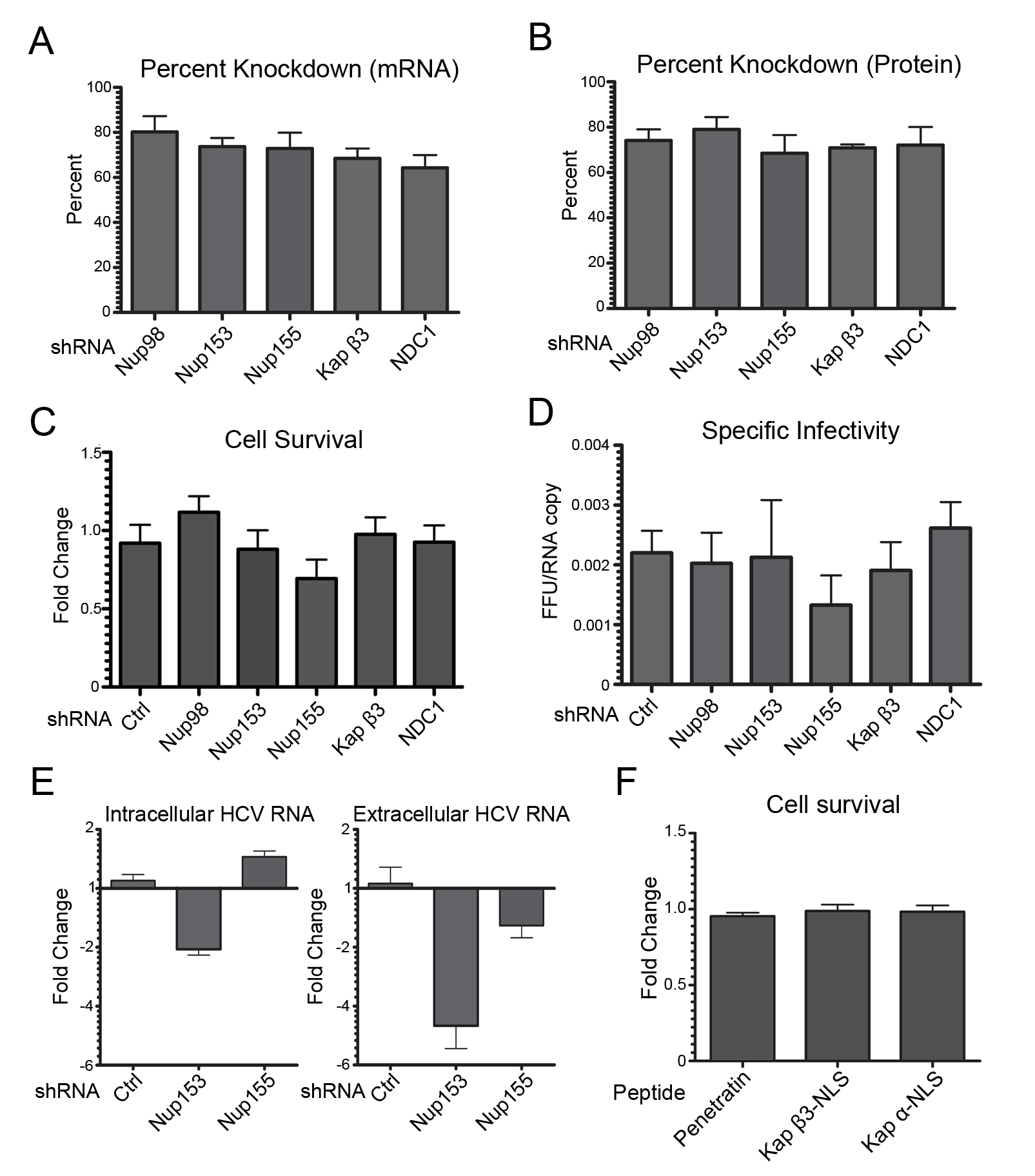

Supplement: Figure S9 — shRNA-mediated knockdown of Nups and Kaps and their effects on cell survival and the specific infectivity of HCV. A–D) Huh7.5 cells were coinfected with HCV and lentivirus encoding shRNAs directed against Nup98, Nup155, Nup153, NDC1, Kap β3, or a scrambled control sequence for four days. A) The level of mRNA transcript depletion following shRNA expression was evaluated by qPCR using primers specific for Nup98, Nup153, Nup155, Kap β3, or NDC1. Each sample is normalized to HPRT mRNA levels and expressed as a percent knockdown relative to cells infected with HCV and the lentivirus encoding the scrambled control shRNA sequence. B) Protein depletion following shRNA expression was evaluated by quantitative western blotting using antibodies specific for the indicated proteins. Protein levels were normalized to that of α-tubulin. Fold-change is relative to cells infected with HCV and the lentivirus encoding the scrambled control shRNA sequence. C) The cytotoxic effects of expressing the various shRNA constructs in Huh7.5 cells were evaluated using an MTT cell viability assay. Values for the survival of cells depleted of the indicated proteins represent fold change relative to cells not infected with lentivirus. D) The specific infectivity of HCV particles produced from cells coinfected with lentivirus was assessed by first determining total number of HCV RNA copies/mL in the culture supernatant for each sample. Huh7.5 cells were then infected with identical amounts of HCV RNA copies/mL harvested from each of the coinfected cells and focus-forming units were determined using indirect immunofluorescence microscopy. Values represent focus-forming units per copy of viral RNA. E) Huh7.5 cells were coinfected with HCV and lentivirus encoding a scrambled control sequence or an shRNA directed against a secondary site in either Nup155 or Nup153 distinct from that used in the analysis shown in A–D. Four days after infection, the levels of extracellular and intracellular HCV RNA were [file ppat.1003744.s009.tif]

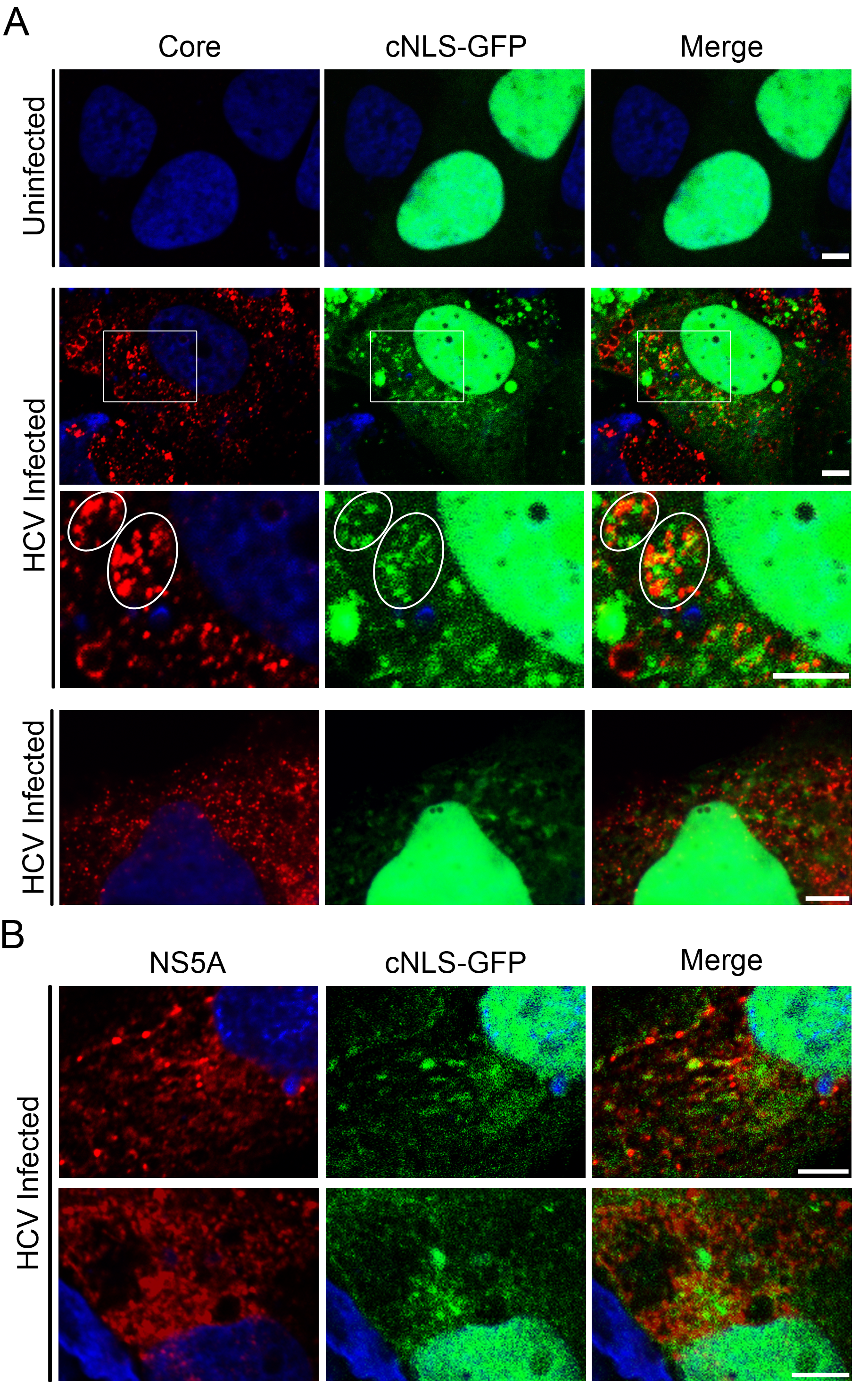

Supplement: Figure S10 — Localization of cNLS-GFP reporter protein to the membranous web. Huh7.5 cells were infected with HCV. Two days following infection, cells were transfected with a plasmid-born construct encoding a cNLS-GFP reporter. Four days post infection, the subcellular localization of the cNLS-GFP protein was examined by fluorescence confocal microscopy (green). The localization of the cNLS-GFP fluorescence signal was compared to that of HCV core (red, panel A) or NS5A (red, panel B) by indirect immunofluorescence confocal microscopy using specific antibodies. In panel A, boxed areas in the second rows of images are shown at higher magnification in the third row. Regions enriched for both the cNLS-GFP reporter and HCV core are highlighted in third row with white ovals. In all panels, uninfected Huh7.5 cells producing the cNLS-GFP reporter are also shown. DNA is stained with DAPI (blue). Scale bars, 5 µm. (TIF) [file ppat.1003744.s010.tif]

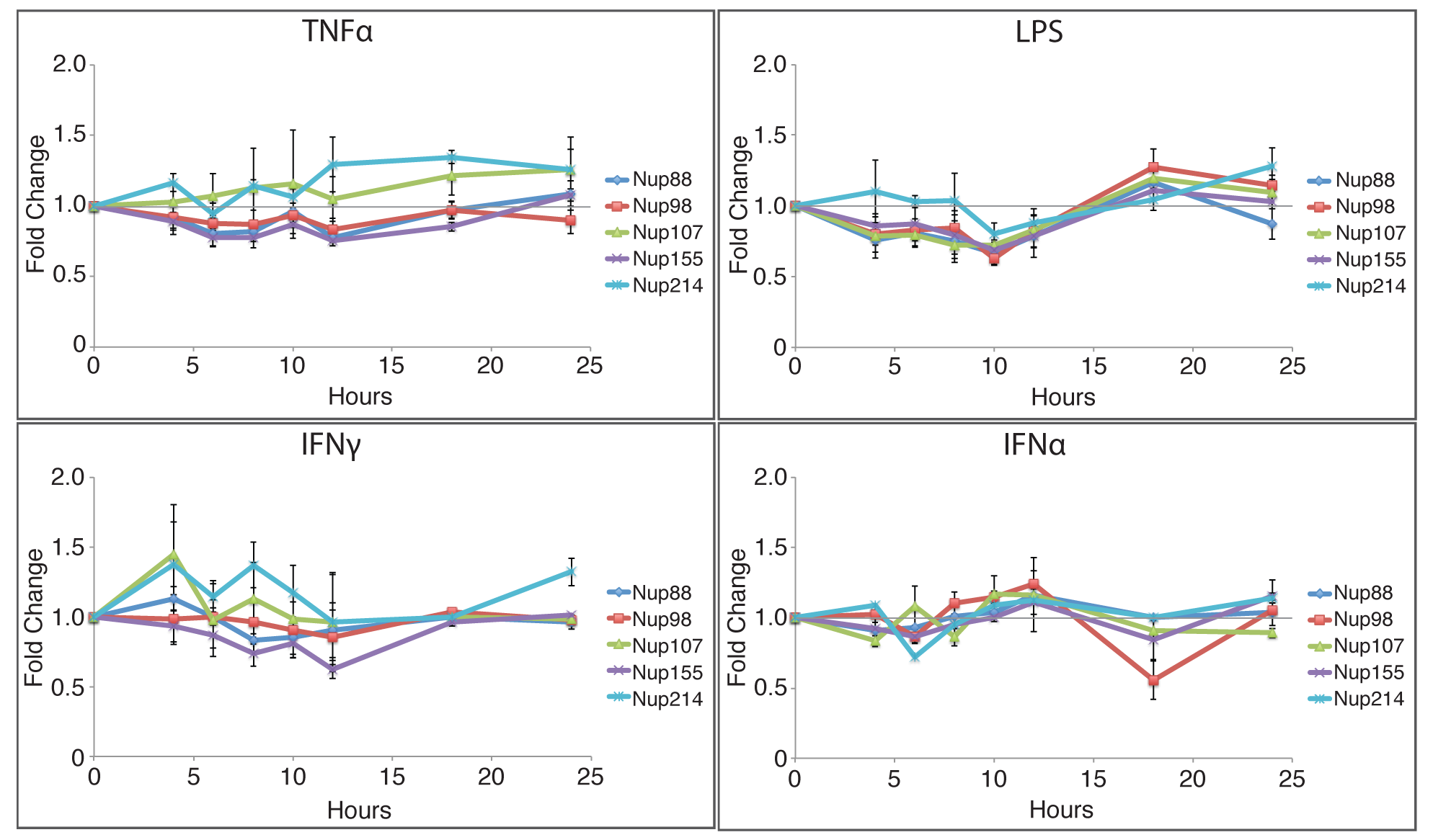

Supplement: Figure S11 — Activation of innate immune pathways does not alter levels of various Nups in Huh7.5 cells. Huh7.5 cells were treated with IFNγ (500 units/mL), TNF-α (100 ng/mL), LPS (0.1 µg/mL), or IFNα (1000 units/mL). At various time points transcript levels of the indicated Nups were evaluated by qPCR. Samples were normalized to HPRT transcript levels and fold change is relative to samples derived from untreated cells. (TIF) [file ppat.1003744.s011.tif]
