# Supplementary material for: Hepatitis C Virus-Induced Cytoplasmic Organelles Use the Nuclear Transport Machinery to Establish an Environment Conducive to Virus Replication
Source: PLoS Pathog. 2013 Oct 31;9(10):e1003744. doi: 10.1371/journal.ppat.1003744 (PMC3814334; doi:10.1371/journal.ppat.1003744)
Supplement: Table S1 — List of real time qPCR primers used in this study. (DOC) [file ppat.1003744.s012.doc]

**Table S1. Real time qPCR primers used in this study**

|  | **Primer Name** | **Sequence** |
| --- | --- | --- |
| 1 | Nup53 Forward | 5'-tcctggaacagggcaaagta-3' |
| 2 | Nup53 Reverse | 5'-tccaactgggcaggagataa-3' |
| 3 | Nup62 Forward | 5'-gtggctccagctaccacatc-3' |
| 4 | Nup62 Reverse | 5'-ggctgaattccctgctgag-3' |
| 5 | Nup88 Forward | 5'-ggaaagctgttgggtccatt-3' |
| 6 | Nup88 Reverse | 5'-gggacacagggtaagcagagta-3' |
| 7 | Nup98 Forward | 5'-accacccagaacactggctt-3' |
| 8 | Nup98 Reverse | 5'-ggctgtgaggcttgggttac-3' |
| 9 | Nup107 Forward | 5'-gagcgccacaaactgtacct-3' |
| 10 | Nup107 Reverse | 5'-tgggtcaagtccctggtcta-3' |
| 11 | Nup153 Forward | 5'-agcctgtgaaacaccgaaac-3' |
| 12 | Nup153 Reverse | 5'-agctggaagatgaagcagtca-3' |
| 13 | Nup155 Forward | 5'-ctccactgctgcctgtgata-3' |
| 14 | Nup155 Reverse | 5'-cggaagagtggttggaaatc-3' |
| 15 | Nup214 Forward | 5'-gctccgcctttacaaacaga-3' |
| 16 | Nup214 Reverse | 5'-cacaggctttccaggtcact-3' |
| 17 | Nup358 Forward | 5'-tgcaactactggcccttca-3' |
| 18 | Nup358 Reverse | 5'-catagactgggccctttgtg-3' |
| 19 | Nup205 Forward | 5'-caggcagaggatcgacaact-3' |
| 20 | Nup205 Reverse | 5'-gcgaccacaggcattaactc-3' |
| 21 | NDC1 Forward | 5'-catttgcagaagggtcagatg-3' |
| 22 | NDC1 Reverse | 5'-tcaggtcctgcaaggctaaa-3' |
| 23 | Kap β3 Forward | 5'-taatgccgtgggacagatg-3' |
| 24 | Kap β3 Reverse | 5'-ccttggtcttccatggtctg-3' |
| 25 | IRF-1 Forward | 5'-ggattccagccctgatacct-3' |
| 26 | IRF-1 Reverse | 5'-cctgcatgtagcctggaact-3' |
| 27 | HPRT Forward | 5'-cctggcgtcgtgattagtg-3' |
| 28 | HPRT Reverse | 5'-acaccctttccaaatcctcag-3' |
| 29 | HCV Forward | 5'-tctgcggaaccggtgagta-3' |
| 30 | HCV Reverse | 5'-gtgtttcttttggtttttctttgaggtttagg-3' |
| 31 | HCV probe | 5'-FAM-cacggtctacgagacctcccggggcac-TAMARA-3' |
| 32 | Kap α1 Forward | 5'-tgttggctctccttgcagtt-3' |
| 33 | Kap α1 Reverse | 5'-ttcttgttgcggcaaagatt-3' |
| 34 | Kap α7 Forward | 5'-cttgctgggccctttcttat-3' |
| 35 | Kap α7 Reverse | 5'-tgtgcatcagcagctctacc-3' |
| 36 | Kap β1 Forward | 5'-atgcgaagggagcactacag-3' |
| 37 | Kap β1 reverse | 5'-gggttccagtcatcgtcatc-3' |
