# Supplementary material for: Hepatitis C Virus-Induced Cytoplasmic Organelles Use the Nuclear Transport Machinery to Establish an Environment Conducive to Virus Replication
Source: PLoS Pathog. 2013 Oct 31;9(10):e1003744. doi: 10.1371/journal.ppat.1003744 (PMC3814334; doi:10.1371/journal.ppat.1003744)
Supplement: Table S2 — List of the shRNA sequences used to deplete Nups or Kaps in this study. (DOC) [file ppat.1003744.s013.doc]

Table S2. Lentiviral shRNA sequences used in this study

| **Gene** | **Sequence** |
| --- | --- |
| Scrambled Control | CCGGGCGCGATAGCGCTAATAATTTCTCGAGAAATTATTAGCGCTATCGCGCTTTTTG |
| Nup98 | CCGGCCCTTGCAGATGGCTCTTAATCTCGAGATTAAGAGCCATCTGCAAGGGTTTTTG |
| Nup153 | CCGGTCTGCTGGTGGTGGCATATTTCTCGAGAAATATGCCACCACCAGCAGATTTTTG |
| Nup155 | CCGGCCCTATCCAAATCCATCCTTTCTCGAGAAAGGATGGATTTGGATAGGGTTTTTG |
| Kapβ3 | CCGGCCATCACTGAAGCACATCGTTCTCGAGAACGATGTGCTTCAGTGATGGTTTTTTG |
| NDC1 | CCGGCCTGTATAGTTCCTATGTAATCTCGAGATTACATAGGAACTATACAGGTTTTTG |
| Nup153-2 | CCGGGCTACAAAGATACTTCAACAACTCGAGTTGTTGAAGTATCTTTGTAGCTTTTTG |
| Nup155-2 | CCGGGCTCTTTAGTATTGCCCTTTACTCGAGTAAAGGGCAATACTAAAGAGCTTTTTG |
