# Supplementary material for: Hepatitis C Virus-Induced Cytoplasmic Organelles Use the Nuclear Transport Machinery to Establish an Environment Conducive to Virus Replication
Source: PLoS Pathog. 2013 Oct 31;9(10):e1003744. doi: 10.1371/journal.ppat.1003744 (PMC3814334; doi:10.1371/journal.ppat.1003744)
Supplement: Text S1 — Supplementary Materials and Methods . (DOC) [file ppat.1003744.s014.doc]

**EXTENDED MATERIALS AND METHODS**

**Quantitative real-time PCR (qPCR).**

For analysis of intracellular RNA transcript levels, total RNA was extracted from cells using Trizol (Invitrogen, 15596018) and cDNA was synthesized using random primers (Invitrogen, 48190-011) and superscript II (Invitrogen, 18064014) according to the manufacturers specifications. Primers for qPCR were designed using Primer3 software and primer sequences are supplied in Table S1. PCR efficiency for each primer was determined using the slope of a standard curve derived from qPCR analysis of cDNA serial dilutions. qPCR was done using a SYBR green super mix (Quanta, 95070-500) on a Stratagene Mx3005p real time PCR machine. To obtain the relative abundance of specific RNAs from each sample, ct values were corrected for the specific PCR efficiency of the primer, and normalized to hypoxanthine phosphoribosyltransferase 1 (HPRT) transcript levels. For analysis of extracellular HCV RNA levels, RNA was isolated from 200 μL of the media obtained from infected cells using a High Pure Viral Nucleic Acid Kit (Roche, 11858874001). The cDNA was synthesized using superscript III (Invitrogen, 18080044) and an HCV specific primer (see HCV reverse primer sequence Table S1). qPCR was done using TaqMan Master Mix with HCV primers and labeled probe (Table S1). Graphs representing data obtained from qPCR were constructed using GraphPad Prism 5 software, and statistical analysis of values was done using students t-tests.

**Antibodies**

Antibodies directed against Nup155, NDC1, Nup98 [1], Nup53 [2], Lamin B [3], Nup358/RanBP2 [4], Nup153 [5], HCV NS5A [6] and HCV NS5B [7] have been previously described. Commercially available antibodies were used to detect Nup62, Nup153, Nup214, and Nup358 (mAb414 Covance, MMS-120p), α-tubulin (Sigma-Aldrich, T6074), mouse anti-HCV core (Thermo Scientific, MA1-080), rabbit anti-HCV core (Abcam, ab58713), HCV NS3 (Millipore, MAB8691), karyohperin β3 (Santa Cruz Biotechnology, sc-84578), karyopherin α1/6 (Santa Cruz Biotechnology, sc-6918), FACL4 (Abcam, ab155282), VDAC1 (Abcam, ab15895), Ran (Abcam, ab4781) and the V5 epitope (Abcam, ab27671). For western blotting, the HRP-conjugated and fluor-conjugated secondary antibodies used to detect primary antibodies included: donkey anti–rabbit IgG-HRP (GE Healthcare, NA934V), sheep anti–mouse IgG-HRP (GE Healthcare, NA931V), Alexa Fluor 750 goat anti-rabbit IgG (Invitrogen, A21039), and Alexa Fluor 680 goat anti-mouse IgG (Invitrogen, A21057). For indirect immunofluorescence microscopy, primary antibodies were detected using Alexa Fluor 488 donkey anti-rabbit (Invitrogen, A21206), Alexa Fluor 488 donkey anti-mouse (Invitrogen, A21202), Alexa Fluor 594 donkey anti-mouse (Invitrogen, A21203), Alexa Fluor 594 donkey anti-rabbit (Invitrogen, A11012), Alexa Fluor 594 donkey anti-goat (Invitrogen, A11058) and Alexa Fluor 647 Goat anti-mouse (Invitrogen, A21236) secondary antibodies.

**Western Blotting**

Cells were lysed using SDS-sample buffer, followed by sonication and denaturation at 95°C for 10 min. 10 µg of total protein from each sample was resolved by SDS-PAGE and transferred to nitrocellulose. Membranes were blocked with PBS-T (PBS containing 0.1% Tween-20) containing 5% skim milk for 2 hours at room temperature, followed by incubation with anti-sera containing primary antibodies overnight at 4°C. Following incubation with primary antibodies, membranes were washed and incubated for 2 hours with either the HRP-conjugated or fluor-conjugated secondary antibodies described above. For HRP-conjugated secondary antibodies, membranes were washed in PBS-T followed by initiation of HRP chemiluminescence with ECL detection reagent (GE Healthcare, RPN2106) and the signal was detected using Fuji RX film (Fujifilm, 47410 08399). For fluorescent labeled blots, membranes were sequentially washed in PBS-T, PBS and H_2_O and secondary antibodies were detected with an Odyssey infrared imaging system (Licor). Quantification of protein levels detected with the Licor system was done using Odyssey V3.0 software.

**Immunofluorescence**

Huh7.5 cells grown on glass cover slips were fixed with 3.2% formaldehyde (Sigma, F8775-500ML) at room temperature for 10 minutes then permeablized in 0.2% Triton X-100 (VWR, CA97062-208) for 2 minutes. To visualize lipid droplets, samples were incubated with BODIPY 493/503 (Invitrogen, D3922) for 15 minutes after fixation. Cover slips were then blocked in 2.5% skim milk for 2 hrs and incubated with the indicated primary antibodies at 4°C overnight. Samples were then washed and incubated with secondary antibodies for 45 minutes at room temperature, followed by mounting onto microscope slides using Dapi-Fluoromount-G (Southern BioTech, 0100-20). Epifluorescence images were obtained with an Axio Observer Z1 microscope (Carl Zeiss, Inc.) using a 63x/1.40 NA Oil UPlanS-Apochromat objective lens (Carl Zeiss Inc.). Images taken on the epifluorescent microscope were deconvolved using Axiovision software (Carl Zeiss, Inc.). Confocal images were obtained with a LSM 710 Axio Observer microscope (Carl Zeiss Inc.) using a 63x/1.40 NA Oil DIC Plan-Apochromat objective. Images were acquired as a z-stack series (with a distance of 0.24μm between slices) and are represented as a z-projection. Z-projection and merging of channels was done using ImageJ software (National Institutes of Health). Photoshop 5.0 (Adobe) software was used to adjust brightness and contrast levels for individual images and assemble images into figures.

**Subcellular fractionation**

Huh7.5 cells were infected with HCV as described above. On day 4 after infection cell were removed from plates with trypsin and lysed in isolation buffer (IB)(225 mM Manitol, 75 mM Sucrose, 30 mM Tris-HCL, 0.1 mM EGTA) using a Balch homogenizer. The nuclear fraction was isolated from the lysate by low speed centrifugation at 600 x g. Crude mitochondria and associated membranes were pelleted from the supernatant by centrifugation at 7,000 x g 2 times for 10 minutes each. The supernatant was collectet and the microsomal fraction was isolated from the supernatant by centrifugation at 150,000 x g followed by resuspension of the microsomal pellet in mitochondrial resuspension buffer (MRB)(250mM Mannitol, 5mM HEPES). The mitochondria and associated membranes in the pellet were resuspended in MRB and layered onto 10mL of Percoll Isolation Medium (PIM) (225 mM Mannitol, 25 mM HEPES, 1 mM EGTA, 30% v/v Percoll). Samples were then centrifuged at 95,000 x g for 30 minutes to separate the MAM fracrtion from the mitochondrial fraction. The MAM fraction was isolated from the gradient and centrifuged for 10 minutes at 6,300 x g and the resulting supernatant (containing the MAM) was diluted to 10mL with MRB. These samples were then centrifuged for 60 minutes at 100,000 x g to pellet the MAM fraction. The pellet containing the MAM fraction was resuspended in MRB. The mitochondria containing fraction (isolated from the Percoll gradient) was further purified by centrifugation for 10 minutes at 6,300 x g. The pellet was resuspended in IB and centrifuged again at 6,300 x g for 10 minutes to pellet the purified mitochondrial. Purified mitochondrial fraction was resuspended in MRB. The total amount of protein was calculated for each sample with a DC protein assay kit (BioRad, 500-0116) using the manufacturers protocol. SDS sample buffer was added to each fraction and protein contents were evaluated by western blotting.

**Immunoprecipitation**

HEK293T cells were transfected with the constructs encoding for HCV proteins described above. 48 hours after transfection, cells were lysed with ice-cold lysis buffer (10 mM Tris-HCl pH 7.5, 150 mM NaCl, 2 mM EDTA, 0.1% Triton-X100, 1 mM PMSF, 2 µg/ml aprotinin, 2 µg/ml leupeptin and 0.1 units/µl RNasin) and incubated on ice for 15 min. Lysates were clarified by centrifugation at 14,000 rpm for 15 minutes at 4˚C. The resulting supernatant was incubated with anti-V5 antibodies for 4 hours at 4°C and then overnight with protein G sepharose beads. Beads were washed 8 times with wash buffer (50 mM Tris-HCl pH7.5, 150 mM NaCl, 0.05% Triton-X100). After each wash, the beads were collected by centrifugation (5000 rpm for 5 minutes at 4°C) and the supernatant was removed. Following the last wash, the supernatant was removed and protein was stripped from the beads by adding SDS-sample buffer and incubation at 95°C for 10 minutes. For the immunoprecipitation experiments done in HCV infected cells, Huh7.5 cells were infected for 4 days. Cells were then lysed with a Balch homogenizer in IB (225 mM Manitol, 75 mM Sucrose, 30 mM Tris-HCL, 0.1 mM EGTA) and the nuclear fraction was removed from the lysate by low speed centrifugation (600 x g for 5 minutes). The remaining cytoplasmic fraction was incubated with mAb414 antibodies for 4 hours at 4°C and then overnight with protein G Dynabeads (Novex, 10004D). Beads were washed 8 times with wash buffer (50 mM Tris-HCl pH7.5, 150 mM NaCl, 0.05% Triton-X100). Following the last wash, the supernatant was removed and protein was stripped from the beads by adding SDS-sample buffer and incubation at 95°C for 10 minutes. Samples were analyzed by western blotting.

**Synthetic peptides**

Peptides were manufactured and purified to at least 85% purity by GL Biochem Ltd (Shanghai, China). For immunoprecipitation experiments, 125 µM of the indicated peptide was added to the culture medium 12 hours post transfection. To assess the effects of peptide treatments on HCV replication, 125 µM of the indicated peptide was added to HCV infected Huh7.5 cells 4 hours after infection and again 2 days after infection. HCV levels were determined 4 days after infection by qPCR of viral RNA.

**SUPPLEMENTAL REFERENCES**

1. Mitchell JM, Mansfeld J, Capitanio J, Kutay U, Wozniak RW (2010) Pom121 links two essential subcomplexes of the nuclear pore complex core to the membrane. J Cell Biol 191: 505-521.

2. Hawryluk-Gara L, Shibuya E, Wozniak R (2005) Vertebrate Nup53 interacts with the nuclear lamina and is required for the assembly of a Nup93-containing complex. Mol Biol Cell 16: 2382-2394.

3. Chaudhary N, Courvalin JC (1993) Stepwise reassembly of the nuclear envelope at the end of mitosis. J Cell Biol 122: 295-306.

4. Joseph J, Liu ST, Jablonski SA, Yen TJ, Dasso M (2004) The RanGAP1-RanBP2 complex is essential for microtubule-kinetochore interactions in vivo. Curr Biol 14: 611-617.

5. Bodoor K, Shaikh S, Salina D, Raharjo WH, Bastos R, et al. (1999) Sequential recruitment of NPC proteins to the nuclear periphery at the end of mitosis. J Cell Sci 112 ( Pt 13): 2253-2264.

6. Lindenbach BD, Evans MJ, Syder AJ, Wolk B, Tellinghuisen TL, et al. (2005) Complete replication of hepatitis C virus in cell culture. Science 309: 623-626.

7. Wilson JA, Jayasena S, Khvorova A, Sabatinos S, Rodrigue-Gervais IG, et al. (2003) RNA interference blocks gene expression and RNA synthesis from hepatitis C replicons propagated in human liver cells. Proc Natl Acad Sci 100: 2783-2788.
